# Bimolecular photoinduced symmetry-breaking charge separation of perylene in solution

**DOI:** 10.1007/s43630-023-00504-3

**Published:** 2023-12-22

**Authors:** Johannes Wega, Eric Vauthey

**Affiliations:** Department of Physical Chemistry, University of Geneva, Quai Ernest Ansermet 30, 1205 Geneva, Switzerland

**Keywords:** Symmetry-breaking charge separation, Perylene, Bimolecular, Photoinduced electron transfer, Room-temperature ionic liquids

## Abstract

**Abstract:**

Photoinduced symmetry-breaking charge separation (SB-CS) results in the generation of charge carriers through electron transfer between two identical molecules, after photoexcitation of one of them. It is usually studied in systems where the two reacting moieties are covalently linked. Examples of photoinduced bimolecular SB-CS with organic molecules yielding free ions remain scarce due to solubility or aggregation issues at the high concentrations needed to study this diffusion-assisted process. Here we investigate the excited-state dynamics of perylene (Pe) at high concentrations in solvents of varying polarity. Transient absorption spectroscopy on the subnanosecond to microsecond timescales reveal that self-quenching of Pe in the lowest singlet excited state leads to excimer formation in all solvents used. Additionally, bimolecular SB-CS, resulting in the generation of free ions, occurs concurrently to excimer formation in polar media, with a relative efficiency that increases with the polarity of the solvent. Moreover, we show that SB-CS is most efficient in room-temperature ionic liquids due to a charge-shielding effect leading to a larger escape of ions and due to the high viscosity that disfavours excimer formation.

**Graphical abstract:**

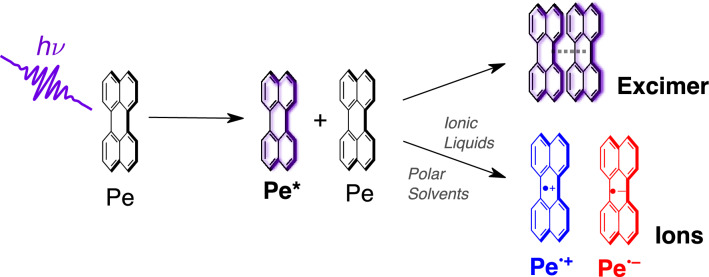

**Supplementary Information:**

The online version contains supplementary material available at 10.1007/s43630-023-00504-3.

## Introduction

Photoinduced symmetry-breaking charge separation (SB-CS) can be described as an electron transfer between two identical molecules after optical excitation of one of them, resulting in the generation of radical ions [1, 2]:

1 $$\begin{aligned} {\text {M}}^* + {\text {M}}&\xrightarrow {{\text {SB-CS}}} {\text {M}}^{\cdot +} + {\text {M}}^{\cdot -}. \end{aligned}$$

It enables the conversion of light into free charge carriers with minimal thermal losses, and could, thus, be used in a wide range of applications including photovoltaics and artificial photosynthesis [3–6].

**Fig. 1:**
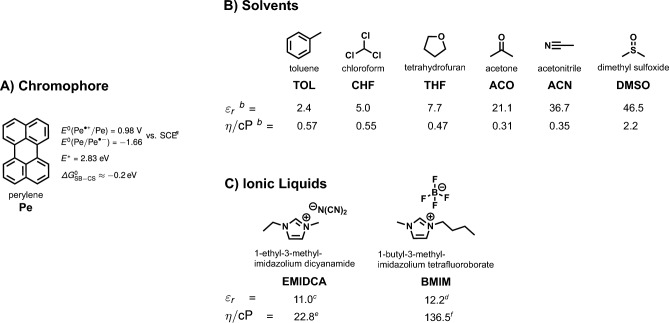
**A** Chemical structure, electronic and redox properties of perylene. **B** Dipolar solvents used in this investigation together with their dielectric constant $\varepsilon _r$ and viscosity $\eta$ at $20^{\circ }\hbox {C}$. **C** Room temperature ionic liquids used in this study. A more extensive list of solvent properties is given in Tables S1 and S2 (ESI). (a) Ref. [7] (b) Ref. [8] (c) Ref. [9] (d) Ref. [10] (e) Ref. [11] (f) Ref. [12]

The number of molecules that may undergo SB-CS is, however, limited. The free energy gain upon SB-CS ($\Delta G_{{\text {SB-CS}}}^0$) may be estimated using the Weller equation [13]:

2 $$\begin{aligned} \Delta G_{{\text {SB-CS}}}^0= & {} e\left[ E^{0}\left( \textrm{M}^{\cdot +} / \textrm{M}\right) -E^{0}\left( \textrm{M} / \textrm{M}^{\cdot -}\right) \right] \nonumber \\{} & {} - E^* + U(r), \end{aligned}$$

whereby $E^{0}\left( \textrm{M}^{\cdot +} / \textrm{M}\right)$ and $E^{0}\left( \textrm{M} / \textrm{M}^{\cdot -}\right)$ are the oxidation/reduction potentials of the chromophore, $E^*$ its excited state energy and *U*(*r*) the electrostatic potential energy gained upon generating the ion pair at a distance *r*. In the limit of infinite dilution or zero ionic strength, this term can be approximated by Coulomb’s law [13]:

3 $$\begin{aligned} U(r) = C = - \frac{e^2}{4 \pi \varepsilon _{\textrm{r}} \varepsilon _0 r}, \end{aligned}$$

with *e* being the elementary charge, $\varepsilon _0$ the vacuum permittivity and $\varepsilon _{\textrm{r}}$ the dielectric constant of the solvent. Considering the excited state to result from a one-electron HOMO $\rightarrow$ LUMO excitation, the excited-state energy $E^*$ can be crudely approximated as the HOMO-LUMO gap. As the difference in redox potentials also corresponds to this gap, $\Delta G_{{\text {SB-CS}}}^0$ should essentially be zero due to the cancellation of the first two terms in Eq. 2. However, the Coulomb and exchange contributions to the excited-state energy [14] can lead to a significant driving force for SB-CS, especially in polar solvents where the ion pair state is stabilised. Because of this, photoinduced SB-CS and has been observed with a variety of chromophoric systems [15–21].

In most of these studies, however, SB-CS is intramolecular with the chromophoric sub-units covalently linked. Reports of inter/bimolecular SB-CS on the other hand remain scarce [22]. Assuming reaction 1 to be limited by diffusion with a rate constant of $k_{{\text {diff}}} \approx 2\times 10^{10}\,\hbox {M}^{-1} \hbox {s}^{-1}$ in e.g. acetonitrile and a typical excited state lifetime of an organic chromophore of $\tau _0 \approx 5\,\hbox {ns}$ highlights that the concentration needed to quench half of the excited states is in the millimolar regime. As these concentrations are much larger than those normally used in photophysical studies, intermolecular SB-CS is in many cases irrelevant. On the other hand, the energy of the longer-lived triplet excited state is too low for SB-CS to occur according to Eq. 1. However, the formation of radical ions upon triplet-triplet annihilation has been reported in polar environments [23–25]. The exact mechanism of this process, i.e. whether it involves the population of the singlet excited state followed by SB-CS as in Eq. 1, is still unclear.

The limited solubility and the propensity to aggregate of many organic chromophores complicate the study of bimolecular photoinduced SB-CS. One suitable candidate is perylene (Pe, Fig. 1A) due to its intrinsic electronic and redox properties, which render SB-CS slightly exergonic ($\Delta G_{{\text {SB-CS}}}^0 \approx -0.2\,\hbox {eV}$ neglecting *U*). Indeed, multichromophoric systems undergoing intramolecular SB-CS based on Pe [26–28] or perylene diimide (PDI) [15, 29–31] chromophores have been extensively studied in the past. Although CS is typically observed in polar solvents, Giaimo et al. [15] observed SB-CS in a PDI-dimer even in the low polarity solvent toluene.

Other processes, like excimer formation, are often observed in these multichromophoric systems. As they compete with CS, they are sometimes termed "parasitic" [2]. Aster et al. [28] could demonstrate how SB-CS can be systematically tuned by controlling the coupling between the chromophores and thus inhibiting excimer formation. On the hand, Sung et al. [32] showed that the excimer of a PDI-dimer could actually mediate SB-CS.

Given that mutual orientation and distance cannot be controlled in intermolecular processes, excimer formation can, in principle, not be prevented. However, an excimer can generally be described as a superposition of a Frenkel, $|{\text {M}}-{\text {M}}^* \rangle \pm |{\text {M}}^*-{\text {M}} \rangle$, and a charge transfer (CT), $|{\text {M}}^{\cdot -}-{\text {M}}^{\cdot +} \rangle \pm |{\text {M}}^{\cdot +}-{\text {M}}^{\cdot -} \rangle$, excitonic state [2, 33]. Therefore, the excimer might play a key role in intermolecular SB-CS as an intermediate between the locally excited reactant state, ${\text {M*+M}}$, and the free ion product, ${\text {M}}^{\cdot +}+{\text {M}}^{\cdot -}$.

Using laser flash photolysis, Kawai et al. [34], Grellmann and Watkins [35], as well as Vauthey et al. [36] observed the simultaneous appearance of the $\hbox {Pe}^{\cdot +}$ and $\hbox {Pe}^{\cdot -}$ absorption bands several microseconds after excitation of Pe in acetonitrile, pointing to the occurrence of photoinduced bimolecular SB-CS. However, no excimer could be observed in these experiments. More recently, Katoh et al. [33] studied the photochemistry of concentrated Pe solutions in toluene and demonstrated self-quenching of Pe by excimer formation using steady-state as well as transient absorption spectroscopy. However, ion formation was not observed. To the best of our knowledge, photoinduced bimolecular SB-CS with Pe was not investigated further since these studies.

Here, we bridge the gap between these earlier investigations by systematically mapping out the excited-state dynamics of Pe at high concentration from subnanosecond to microsecond timescales in six solvents of increasing polarity (Fig. 1B) using transient electronic absorption spectroscopy. We demonstrate that SB-CS is operative and free ions are generated in polar solvents. This process occurs concurrently to the formation of the excimer. We further study the excited-state dynamics of concentrated Pe solutions in two room-temperature ionic liquids (RTIL, Fig. 1C) exploring the influence of high ionic strengths on the efficiency of SB-CS, whereby we demonstrate an increase of the yield of free ions that we attribute to a charge shielding effect.

## Experimental

### Chemicals

Acetonitrile (ACN, Roth, $\ge 99.9\%$), acetone (ACO, Thermo Fisher Scientific, $\ge 99\%$), chloroform (CHF, Thermo Fisher Scientific, $\ge 99.5\%$), dimethylsulfoxide (DMSO, Sigma-Aldrich, $\ge 99\%$), tetrahydrofuran (THF, Thermo Fisher Scientific, $\ge 99.5\%$) and toluene (TOL, Acros Organics, $\ge 99.8\%$), were used as received. Sublimed perylene (Pe, $\ge 99\%$), tris(2,2’-bipyridine)ruthenium(II)-hexafluorophosphate ([Ru(bipy)_3_][$\hbox {PF}_6$]_2_, $\ge 99\%$), *meta*-iodoaniline ($\ge 99\%$) were purchased from Sigma Aldrich and used without further purification. The room-temperature ionic liquids, 1-ethyl-3-methyl- imidazolium dicyanamide (EMIDCA, IoLiTec (Germany), $\ge 98\,\%$) and 1-butyl-3-methylimidazolium tetrafluoroborate (BMIM, Acros Organics, $\ge 98\,\%$) were used as received.

### Steady-state absorption

Stationary electronic absorption spectra were recorded on a Cary 50 spectrophotometer. In order to record the absorption spectra at high concentrations, solutions were measured in a $10\,\upmu \hbox {m}$ pathlength quartz flow cell purchased from Starna Germany. To maximize the transient absorption signals, the highest possible amount of Pe was dissolved in each solvent at room temperature. Subsequently, the solutions were filtered using a $0.4\,\upmu \hbox {m}$ filter to remove any undissolved residue. In order to estimate the saturation concentration, an approximate extinction coefficient of $\varepsilon _{{\text {Pe}}} \approx (38,500\pm4000)\,\hbox {M}^{-1} \,\hbox {cm}^{-1}$ at the absorption maximum of the $\hbox {S}_1 \leftarrow$ $\hbox {S}_0$ absorption band [37, 38] was used. The approximate saturation concentrations found together with the corresponding absorption spectra are shown Fig. S1 (ESI) for each solvent/RTIL.

### Steady-state fluorescence

Stationary fluorescence spectra were measured using a Horiba Fluorolog 322 spectrofluorometer. Raw spectra were corrected using a set of secondary emissive standards [39]. Concentrated solutions were measured in a 1 mm quartz cuvette, whereas dilute solutions were measured in a 1 cm quartz cuvette.

### Time-resolved fluorescence

Time-resolved fluorescence on the nanosecond timescale was measured using a time-correlated single photon counting (TCSPC) setup similar to the one described in Ref. [40]. Briefly, samples were excited at 405 nm using 60 ps pulses at 10 MHz produced by a laser diode (Picoquant, LDH-PC-405). The fluorescence was collected at the front face of the cuvette at magic angle with respect to the excitation employing a Cassegrain type collection optic (Anagrain; Anaspec Research Laboratories Ltd., Berkshire, UK) in $180^{\circ }$ back-scattering geometry and was focused onto a multimode fiber. A Triax-190 (Horiba) imaging spectrograph was then used to disperse the output of the fiber to select the emission wavelength with a bandpass of around 2 nm. The dispersed output of the spectrograph was focused on a single-photon avalanche diode (Micro Photon Devices, MPD-100-CTE) and the TCSPC was performed with a PicoHarp-300 (PicoQuant). The overall instrument response function of the setup is around 200 ps.

Time resolved fluorescence of the concentrated perylene solutions was measured between 435 and 700 nm in steps of 5 nm. In order to reconstruct the time-resolved emission spectrum, each trace was normalised by its area and subsequently multiplied by the intensity of the steady-state fluorescence spectrum at the specific emission wavelength detected in front face geometry.

**Fig. 2:**
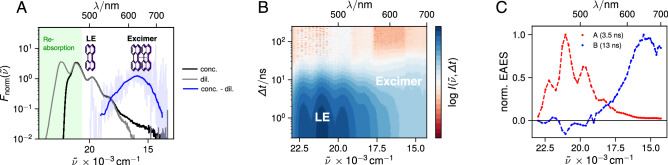
**A** Emission spectra of a concentrated (13 mM, black line) and dilute ($2.5\,\upmu \hbox {M}$, gray line) solution of Pe in TOL normalised at 500 nm. The difference between the two spectra (blue transparent line ) reveals the peak position of the excimer emission. The solid blue line represents a 50-point moving average of the difference spectrum. **B** Time-resolved fluorescence spectra of the concentrated solution measured by TCSPC. **C** Normalised evolution-associated emission spectra (EAES) obtained from global analysis of the data shown in panel (**B**) assuming two successive exponential steps (A $\rightarrow$ B $\rightarrow$)

### Transient Absorption

The sub-nanosecond-microsecond transient absorption (TA) setup was described in detail in Ref. [41]. Excitation was carried out at 355 nm using a passively Q-switched, frequency-tripled Nd:YAG laser (Teem Photonics, Powerchip NanoUV). The laser generated $20\,\upmu \hbox {J}$ pulse of around 300 ps at a repetition rate of 500 Hz. The pump pulses were focused to an approximately $300\,\upmu \hbox {m}^{2}$ spot on the sample. The excitation fluence was attenuated to roughly $4.0\,\hbox {mJ}\,\hbox {cm}^{-2}$, a value at which photoionization of Pe is not expected (see Sect. 1, ESI). The measurements were performed in 1 mm quartz cuvettes under constant bubbling of nitrogen (or compressed air for triplet sensitization experiments, see Section 9, ESI). Due to the high absorbance of the concentrated samples, all probe white light below 480 nm was almost totally absorbed. For this reason, the TA data were cut at 480 nm and the ground-state bleach region was not used for any analysis.

## Results and discussion

### Dipolar solvents

To address any possible aggregation effects, we compared the steady-state absorption spectrum of the concentrated solution ($\sim$ mM) with that of a dilute solution ($\sim \mu\hbox {M}$) for each solvent (Fig. S1, ESI). Apart from a slight red shift of the $\hbox {S}_1\leftarrow \hbox {S}_0$ absorption band at high concentration, that can be attributed mainly to dispersion interactions, no significant difference can be observed. Spano and co-workers [42] demonstrated that the relative intensity of the vibronic absorption bands is prone to change upon aggregation. As no such change is observed here in the saturated solutions, we conclude that Pe does not aggregate significantly in the solvents and at the concentrations used here.

**Fig. 3:**
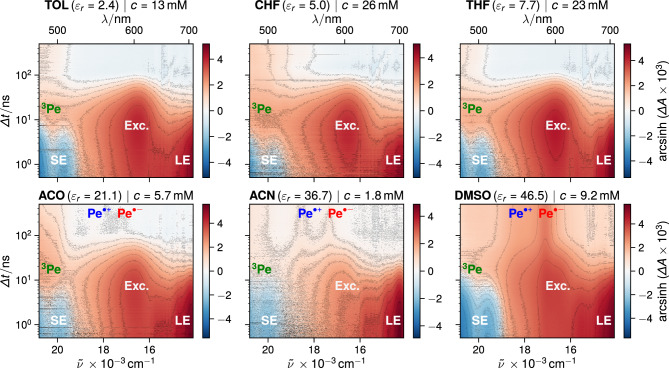
Nanosecond transient absorption measured with concentrated perylene solutions highlighting excimer formation in all solvents and bimolecular SB-CS in the polar solvents. Dielectric constants were taken from Ref. [8]. Note: an arcsinh scale is used as it accentuates weak spectral features relatively to large signals [51, 52]. This scale is hence ideal to contrast the weak ion signals from the intense ESA / excimer bands

On the other hand, we do observe very weak excimer emission red shifted with respect to the Pe monomer emission. Figure 2 A compares the emission spectra normalised at 500 nm of dilute and concentrated solutions of Pe in TOL. The difference below 480 nm is due to the reabsorption of the high-energy side of the emission band in the concentrated solution. Above 570 nm, the concentrated solution exhibits an additional weak emission. Subtraction of the dilute spectrum reveals a broad structureless band peaking around 630–640 nm, which can be assigned to the emission of the Pe excimer, in excellent agreement with literature [33]. We repeated this procedure in the medium polar solvent THF and the polar solvent DMSO and could similarly detect excimer emission in both cases, (see Fig. S2, ESI).

Figure 2 B depicts the time evolution of the fluorescence spectrum measured with the concentrated solution of Pe in TOL. Both the monomer emission as well as the longer-lived excimer emission are visible. Self-quenching is evident by the significantly shorter monomer fluorescence lifetime of about 3.5 ns compared to 5.0 ns at low concentration [37]. Global analysis of the time-resolved emission data assuming a series of two successive exponential steps results in the evolution associated emission spectra (EAES) shown in Fig. 2C. While EAES A is dominated by the monomer emission of Pe and decays in 3.5 ns, EAES B shows a single broad band that coincides with the emission of the Pe excimer. The 13 ns decay time of EAES B is comparable to the 17 ns lifetime of the Pe excimer reported by Katoh et al. [33].^1^

The time evolution of the TA measured with concentrated Pe solutions in different solvents is illustrated in Fig. 3. At early times, the spectral signatures of the $\hbox {S}_{n>1}\leftarrow$ $\hbox {S}_1$ excited state absorption (ESA) of Pe peaking at around 690–710 nm [33, 44, 45] as well as those resulting from $\hbox {S}_1\rightarrow$ $\hbox {S}_0$ stimulated emission (SE) are evident in all solvents. These bands decay concurrently within 3–5 ns, whereas a new absorption band with a maximum around 600–610 nm builds up. In agreement with previous reports [32, 33, 46–49], this band can attributed to the Pe excimer (Exc.).

In the weakly to medium polar solvents TOL, CHF and THF, the excimer band decays entirely within the first 100 ns. However, as the polarity of the solvent increases, the characteristic absorption bands of the radical cation $\hbox {Pe}^{\cdot +}$ around 540 nm [50] and radical anion $\hbox {Pe}^{\cdot -}$ around 580 nm [50] become apparent after the decay of the excimer band. Whereas these bands are rather difficult to distinguish in ACO, they are clearly visible in ACN and DMSO.

**Fig. 4:**
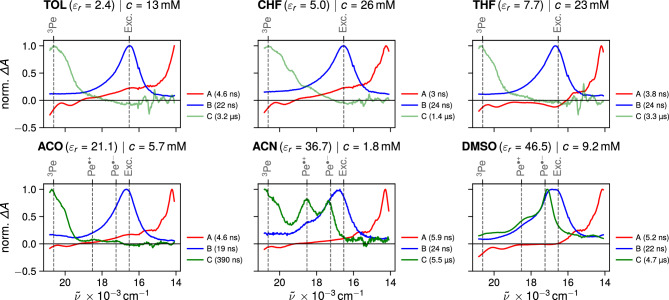
Normalised evolution-associated difference absorption spectra obtained from global analysis of the transient absorption spectra shown in Fig. 3 assuming a series of three successive exponential steps (A $\rightarrow$ B $\rightarrow$ C $\rightarrow$). The gray dashed lines mark the absorption maxima of the species indicated above taken from literature [33, 34, 45, 50]. The EADS C in TOL, CHF, THF, and ACO showed a constant negative amplitude below 15,000 $\hbox {cm}^{-1}$ likely arising from artifacts due to its small signal intensity. Before normalization, these EADS have been shifted by a constant in order to set the amplitude to zero below 15,000 $\hbox {cm}^{-1}$

After the decay of the SE, another positive absorption band is visible in all solvents below 510 nm. The intensity of this band is significantly enhanced in the presence of molecular oxygen (Fig. S7, ESI), which is known to act as triplet sensitisers in certain conditions [53] (see Section 9, SI). As the intrinsic triplet yield of Pe is very small, $\le 0.015$ [54], the exact absorption spectrum of ^3^Pe* is not fully unambiguous [34, 45, 55–57]. To confirm the origin this band below 510 nm, we performed TA measurements of Pe in the presence of a weak electron donor substituted with a heavy atom (see Sect. 9.2, SI). The electron transfer quenching of Pe in the $\hbox {S}_1$ state is followed by an almost quantitative triplet recombination of the ensuing ion pair to ^3^Pe* [58]. The absorption spectrum of ^3^Pe* obtained from these experiments (Fig. S9) coincides with the weak transient band observed below 510 nm with concentrated Pe solutions, confirming its origin. The magnitude of the triplet band relative to the initial intensity of the LE band is very small. It is comparable in all solvents and largest in CHF, mostly likely due to heavy-atom induced intersystem crossing (ISC).

In principle, SB-CS produces the same amount of $\hbox {Pe}^{\cdot +}$ and $\hbox {Pe}^{\cdot -}$, which should mostly decay by charge recombination. As their absorption coefficient at the band maxima are close [45, 50, 59], the $\hbox {Pe}^{\cdot +}$ and $\hbox {Pe}^{\cdot -}$ bands should have similar intensities and decay kinetics. However, the TA data point to a faster decay of $\hbox {Pe}^{\cdot -}$ compared to $\hbox {Pe}^{\cdot +}$ in ACN, ACO and DMSO. This difference can be accounted for by the presence of oxygen, that could not be totally eliminated by nitrogen purging. Indeed, the TA measurements in the presence of molecular oxygen reveal efficient quenching of $\hbox {Pe}^{\cdot -}$, probably by electron transfer (Fig. S7). Morever in DMSO, the $\hbox {Pe}^{\cdot +}$ band is significantly weaker than that of $\hbox {Pe}^{\cdot -}$. This effect can be accounted for by the dimerisation of $\hbox {Pe}^{\cdot +}$ to form ($\hbox {Pe}^{\cdot +})_2$, which is itself characterised by an absorption band with a maximum at 505 nm [59]. The TA spectra in DMSO at long time delays exhibit a band at this wavelength additionally to the $\hbox {Pe}^{\cdot +}$ and $\hbox {Pe}^{\cdot -}$ bands, that could be consistent with the cation dimer. Its formation in DMSO and not in the other polar solvents can be explained by the higher ion concentration in this solvent, due to both a higher Pe concentration and the larger charge separation yield (*vide infra*).

**Fig. 5:**
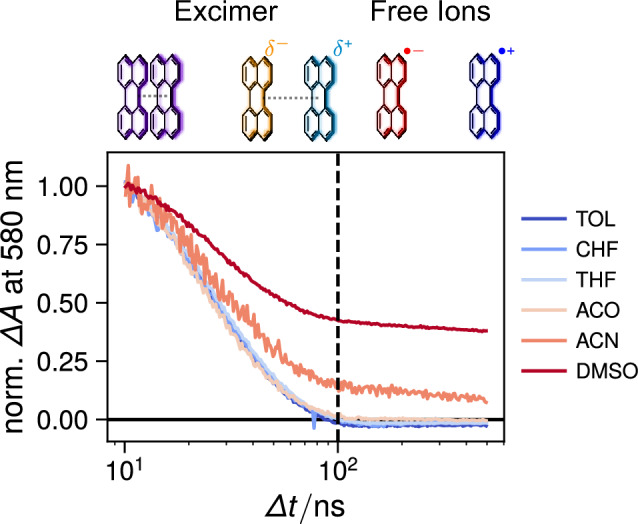
Intensity-normalised time profiles of the transient absorption at 580 nm (absorption maximum of $\hbox {Pe}^{\cdot -}$ [50]) from 10 to 500 ns in different solvents, highlighting the nature of the species contributing to the signal

Global analysis of the TA data assuming a series of three successive exponential steps (A $\rightarrow$ B $\rightarrow$ C $\rightarrow$) resulted in the evolution-associated difference absorption spectra (EADS) and time constants shown in Fig. 4. The non intensity-normalised EADS are depicted in Fig. S3. EADS A contains mostly the spectral features of Pe in the $\hbox {S}_1$ state, as evidenced by the $\hbox {S}_{n>1}\leftarrow$ $\hbox {S}_1$ ESA and SE bands, and decays with a time constant of 3-5 ns. Self-quenching of the excited state is evident as the lifetime of EADS A is decreased compared to the 5–7 ns $\hbox {S}_1$ state lifetime of Pe in dilute solutions [36, 37, 60]. Another indication of this process is the continuous decrease in the lifetime of EADS A with increasing saturation concentration (cf. e.g. CHF and ACN).

EADS B is dominated by a band located around 600 nm in the weakly polar solvents, that can be attributed to the excimer [32, 33, 46–49]. In ACN and DMSO, this band is shifted to shorter wavelength and a shoulder around 540 nm can be observed. EADS B evolves in $\sim 20$–25 ns to EADS C, that does not exhibit the excimer band. This time constant is consistent with the lifetime of the Pe excimer reported in literature [33, 49].

In TOL, CHF and THF, EADS C contains only a band below 550 nm, which decays on the $\upmu$s timescale and can be attributed to the triplet state of Pe as discussed above. From the amplitude of this band relative to that of the $\hbox {S}_{n>1}\leftarrow \hbox {S}_1$ ESA band in EADS A and from their absorption coefficients reported in Ref. [45], the triplet yield can be estimated to be of the order of 3.5, 10 and 5% in TOL, CHF and THF, respectively. This is larger than the intrinsic triplet yield reported for diluted solutions [54], pointing to an additional pathway toward this state, most probably via the excimer. As shown in Fig. S3, the amplitude of EADS C is very small, and its maximum around 500 nm is smaller than the amplitude of EADS B in this region. Because of the small amplitude of the triplet signal, this pathway cannot be confirmed unambiguously.

In polar solvents (ACO, ACN, DMSO), EADS C additionally contains the spectral features of both ions. In ACO, the ion bands are much weaker than the ^3^Pe* band. They have almost the same intensity as the triplet band in ACN and are larger in the more polar DMSO. In the latter solvent, EADS C also exhibits a small maximum above 500 nm that could be attributed to ($\hbox {Pe}^{\cdot +})_2$.^2^ This EADS decays on the microsecond timescale. The intensities of the triplet and ion bands are too small to extract very precises time constants for each of the species contributing to this EADS. Given the microsecond timescale associated with the ion bands, one can conclude that the Pe ions are free ions and should decay non-geminately with a second-order kinetics.

To have a better insight into the efficiency of the SB-CS, we estimated the normalised free ion yield, $\phi _{{\text {FI}}}^n$, from the relative intensity of the $\hbox {Pe}^{\cdot +}$ band at 540 nm (see SI Sect. S7 for details) after normalisation to a quenching efficiency of unity, i.e. $\phi _{{\text {q}}}=1$, to obtain a value independent of Pe concentration. Despite, the crudeness of this approach, the so-obtained $\phi _{{\text {FI}}}^n$ values, listed in Table 1 show a clear increase with the solvent polarity that is beyond the limit of error (section 7.2, SI).

**Table 1:** Estimated free-ion yields, $\phi _{{\text {FI}}}^n$, (normalised to unit quenching efficiency) in different solvents

Solvent	$\phi _{{\text {FI}}}^n / \%$
ACO	$0.3 \pm 0.2$
ACN	$11 \pm 4$
DMSO	$16 \pm 4$
EMIDCA	$55 \pm 23$
BMIM	$46 \pm 26$

The polarity dependence of the ion signal is further highlighted in Fig. 5, which compares the intensity-normalised time profiles of the TA intensity at 580 nm, the absorption maximum of $\hbox {Pe}^{\cdot -}$, in the different solvents. During the first 100 ns, the decay of the excimer is evident in all solvents since the excimer band overlaps with that of $\hbox {Pe}^{\cdot -}$. While the TA signal decays to zero in the non-polar solvents, an almost constant TA signal remains in the polar solvents. It can be attributed to free ions [61, 62], which recombine non-geminately on a longer microsecond timescale.

**Fig. 6:**
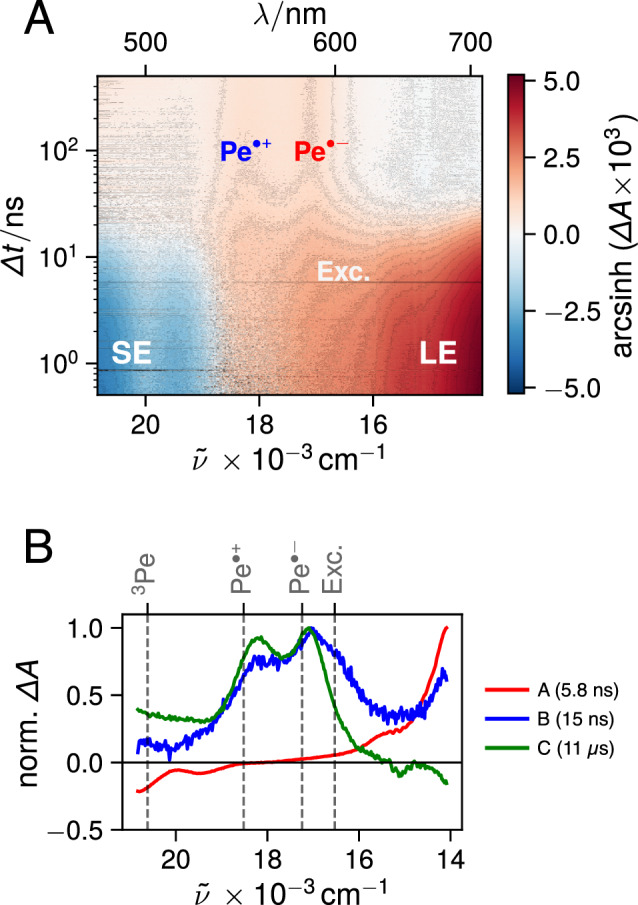
**A** Nanosecond transient absorption measured with Pe in EMIDCA. **B** Normalised evolution-associated difference absorption spectra obtained from global analysis of the transient absorption data assuming a series of three successive exponential steps (A $\rightarrow$ B $\rightarrow$ C). The concentration of Pe was around 3.9 mM

### Ionic liquids

In order to investigate the influence of a high ionic strength, we measured the excited-state dynamics of concentrated Pe solutions in two room-temperature ionic liquids (RTILs, Fig. 1B). The TA spectra and the EADS obtained from subsequent global analysis reveal that SB-CS occurs in both RTILs, despite their relatively high viscosity (Fig. 6). However, contrary to the dipolar solvents, the excimer band is much less pronounced and only appears as a shoulder in EADS B. Due to the high viscosity of these liquids, the diffusion rate constant as estimated by the simple equation:

4 $$\begin{aligned} k_{\text {diff }}=\frac{8 R T}{3 \eta } \end{aligned}$$

with *R* being the ideal gas constant, *T* the temperature, and $\eta$ the viscosity, would be too low to account for any significant diffusional quenching and subsequent ion generation (Table S2, SI). Indeed, several past reports [63–66] have documented quenching rate constants in RTILs that exceed the calculated value of $k_{\text {diff }}$. In fact, the flux of two species towards each other is time-dependent as shown by Schmoluchowski [67] and, consequently, the quenching rate is time dependent as well. The latter varies from its static limit, where quenching occurs without significant diffusion with reactant pairs at short enough distance, to the steady-state limit given by Eq. 4 [68, 69]. Whereas this steady-state limit is rapidly reached, $<1\,\hbox {ns}$, in low viscosity liquids, it may requires several microsecond-milliseconds to be attained in highly viscous liquids (Fig. S6, SI). As a consequence, for chromophores with short excited-state lifetimes (several ns and below), the effective electron transfer quenching rate in highly viscous substances like RTILs may actually be several orders of magnitude larger than that calculated from Eq. 4. Taking these non-stationary effects into account for the RTILS results in a diffusion rate that is indeed larger than predicted by Eq. (4), but still 1–2 orders of magnitude smaller than in the low-viscosity solvents (see Table S3, SI). This is also evidenced in the global analysis by the weakness of the excimer and ion bands (*cf. eg.* Fig. 6B) as less excited states are quenched diffusionally (Table S3, ESI).

**Fig. 7:**
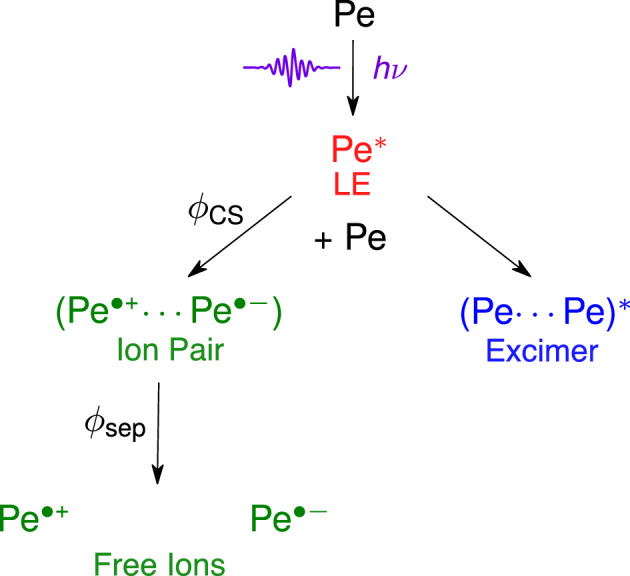
Reaction scheme used for the global target analysis of the transient absorption data. The intrinsic decay of $\hbox {Pe}^*$ to the ground state was also included but is not shown here. The population of ^3^ $\hbox {Pe}^*$ and the charge recombination of the geminate ion pair, also not shown here, were not included

### Mechanism of free ion formation

Although the EADS obtained from a global analysis assuming successive exponential steps allows for a visualisation of the spectral changes, they can not necessarily be assigned to a single species or state [70, 71]. This is clearly the case here for EADS B in dipolar environments, which contains both excimer and ions features. In the RTILs, EADS B exhibits almost only ion bands. This points to a pathway for free ion formation that is concurrent to excimer formation. If the ions were generated from excimer dissociation, the excimer lifetime in polar solvents should be significantly shorter than in weakly polar solvents, contrary to the observation in ACN and DMSO. To test this, we performed global target analysis with a model assuming two parallel quenching pathways, one leading to the excimer and the other to the ions (Fig. 7). The species-associated difference absorption spectra (SADS) obtained from such target analysis in DMSO are illustrated in Figure S10. Now the SADS of the excimer resembles the excimer spectrum measured in weakly polar solvents with a maximum at 600 nm. The agreement is also better in ACN, although a weak shoulder at the $\hbox {Pe}^{\cdot +}$ maximum is still visible in the excimer SADS.

A major shortcoming of this target scheme is that it does not include geminate charge recombination (CR), i.e. it assumes all the ions pairs generated upon SB-CS evolves towards free ions. CR can be expected to be slow here because it is highly exergonic and should occur in the Marcus inverted region [60]. However, it should probably not be slow enough to assume an escape yield of unity especially in DMSO, which is significantly more viscous than ACN. Therefore, the remaining ion contribution to the excimer SADS could arise from geminate ion pairs recombining on a timescale similar to that of the excimer decay. Unfortunately, a target scheme including geminate CR contains too many adjustable variables to allow for a meaningful global analysis of the data. Because of the uncertainty on the fraction of ion pairs that recombine geminately, quantitative estimation of the branching ratio of the excimer and SB-CS pathway cannot be achieved.

**Fig. 8:**
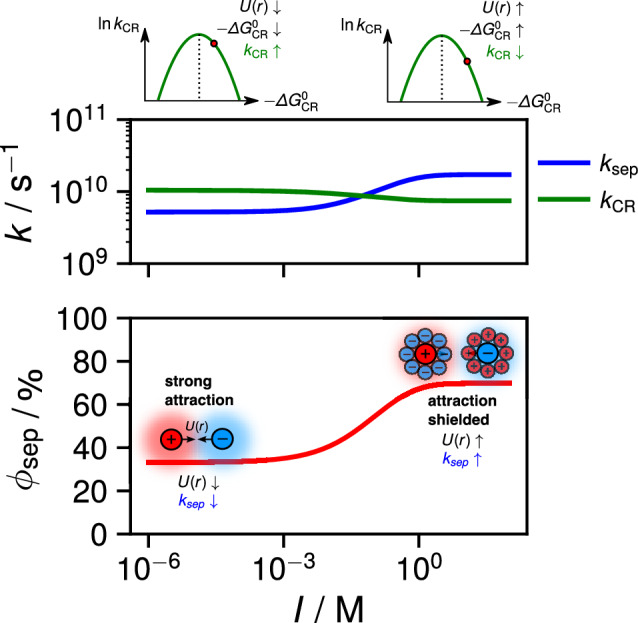
Simulation of the cage-escape yield $\phi _{{\text {sep}}}$ as a function of ionic strength *I* based on a formal kinetic reaction accounting for charge screening assuming spherical ions and the semi-classical Marcus expression for charge recombination (CR). The simulation highlights that $\phi _{{\text {sep}}}$ increases as *I* increases (bottom) due to an increase in diffusional separation rate $k_{{\text {sep}}}$ and a subsequent decrease in CR rate constant $k_{{\text {CR}}}$ caused by the screening of the Coulombic attraction of the ion pair at high ionic strengths

Applying the same target analysis as above to the transient absorption data recorded in RTILs does not result in a SADS corresponding to the excimer (Fig. S10, ESI). Instead, the SADS of the two quenching products are similar and dominated by the ion bands. The main difference is the lifetime, one species decays on the $\upmu$s timescale, as expected for free ions, whereas the other, which contains additionally weak excimer contribution, decays in 17 ns. This latter SADS most probably corresponds to the ion pairs that undergo geminate recombination on the same timescale as excimer decay, while the other SADS corresponds to the fraction that dissociates into free ions. Within this assumption, this fraction amounts to about 20–25% of the total ion-pair population in both RTILs.

From this relatively crude analysis, it appears that a reaction scheme where SB-CS occurs concurrently to excimer formation reproduces the observed population dynamics qualitatively well. A more precise analysis would require taking the time dependence of the quenching rate as well as the non-Markovian nature of charge recombination into account [45, 60, 72]. However, the complication introduced by the involvement of the excimer as well as the small quenching efficiency and the resulting small ion population preclude such an approach.

This reaction scheme with parallel quenching pathway resembles that encountered in a ’conventional’ bimolecular photoinduced ET reaction, where the donor and the acceptor are chemically distinct, and involving the parallel formation of ion pairs and exciplex [73, 74]. In this case, the branching ratio is known to strongly depends on the solvent polarity as also found here. Exciplex formation dominates in non-polar solvents, whereas quenching yields almost exclusively ion pairs in polar environments. The key factor for this behaviour is the balance between the electronic coupling and the driving force, both affecting the ET rate constants. In the normal regime, ET reactions become slower with decreasing driving force. However, a weakly exergonic ET can still be fast enough to quench an excited molecule provided the electronic coupling, *V,* is sufficiently large. This constraint for a large coupling limits the distances and mutual orientations at which quenching can occur, and leads to the formation of a highly coupled product, i.e. an exciplex or a tight ion pair [62]. On the other hand, more exergonic ET can result in efficient quenching even if the reactants are not at a distance/orientation associated with the highest coupling. This leads to less a coupled quenching product, namely loose ion pairs. As the ET driving force increases significantly with solvent polarity, exciplexes are mostly encountered in non- and weakly-polar environments. Similar reasoning can be applied here for SB-CS with Pe. As discussed above, this process is predicted to be weakly exergonic even in polar environments, and is thus probably not operative in non- and weakly-polar media. In this case, diffusional encounter between Pe* and Pe leads to the excimer, which is mainly stabilised by excitonic interactions [33]. In moderately polar solvents, like CHF and THF, SB-CS might be energetically feasible but requiring large coupling to be operative within the excited-state lifetime of Pe. In such a situation, if the ion pairs are formed in parallel to the excimer, they would be so strongly coupled that they should rapidly collapse to the excimer as observed for intramolecular SB-CS [28]. Finally in highly polar environments, SB-CS should be exergonic enough to allow for quenching to occur at distances/orientations where coupling is not optimal. Solvation energy should stabilise the ion pair relative to the excimer, preventing its collapse to the excimer.

In ’conventional’ bimolecular photoinduced ET, ion pairs can also be generated upon exciplex dissociation, provided the solvent is polar enough [73–75]. Here, we have no evidence for the formation of ions from the dissociation of the excimer, although it cannot be totally ruled out. However, contrary to the exciplex, there is no significant charge transfer in an excimer. If excimers were polar, their fluorescence should exhibit a significant solvatochromism, contrary to the observation. Consequently, ion formation formally corresponds to a charge separation within the excimer. According to Katoh et al. [33], the Pe excimer state is located 0.44 eV below the $\hbox {S}_1$ state of Pe. In this case, SB-CS in the Pe excimer is most probably endergonic even in highly polar solvents. Consequently, excimer formation is detrimental to intermolecular SB-CS with Pe.

The normalised free ion yield, $\phi _\textrm{FI}^n$ (Table 1), depends on two factors: (1) the relative efficiency of SB-CS vs. excimer formation, $\phi _\textrm{CS}$, and (2) the relative efficiency of ion pair dissociation vs. geminate charge recombination, $\phi _\textrm{sep}$, i.e. $\phi _\textrm{FI}^n=\phi _\textrm{CS}\phi _\textrm{sep}$. An increase of solvent polarity favours a larger $\phi _\textrm{FI}^n$ by increasing both $\phi _\textrm{CS}$ and $\phi _\textrm{sep}$. However, ion pair dissociation is a diffusive process and, thus, $\phi _\textrm{sep}$, hence $\phi _\textrm{FI}^n$, should tend to zero in highly viscous media. Consequently, the $\phi _\textrm{FI}^n$ values measured in RTILs are surprisingly large in view of the high viscosity and relatively moderate dielectric constant of these liquids (Table S2, ESI). Several factors can be invoked to account for these free ion yields. Despite their not very high dielectric constant, most RTILs behave similarly to highly polar solvents like DMSO when considering electron transfer processes and solvatochromism [76, 77]. Additionally, excimer formation requires efficient diffusion to achieve close contact, whereas SB-CS can occur at a non-optimal distance/orientation. Excimer formation is, thus, less efficient in highly viscous media. These two factors account for the high $\phi _\textrm{CS}$ observed in EMIDCA and BMIM. They should also favour the formation of ion pairs that are not too strongly coupled and, for which CR is thus relatively slow.

In general, the distribution of ion pair structures depends on the reactant pair distribution at the instant of CS. Because of this intrinsic non-Markovian character of bimolecular photoinduced ET reactions [60, 62, 72], there is no simple expression for $\phi _{{\text {sep}}}$. If, for the sake of simplicity, we assume a narrow distribution of ion pairs structures, $\phi _{{\text {sep}}}$ can be estimated as [53, 78–80]:

5 $$\begin{aligned} \phi _{\text {sep }}=\frac{k_{\text {sep }}}{k_{\text {sep }}+k_{\text {CR}}}, \end{aligned}$$

where $k_{\text {sep}}$ is the dissociation rate constant and $k_{\text {CR}}$ the rate constant of charge recombination (CR) of the ion pair. As discussed in detail in the ESI (Sect. S11), $k_{\text {sep}}$ can be viewed as a diffusive escape of the ions from an electrostatic potential well, *U*(*r*), and can estimated using the Eigen equation [81, 82]. While the approximation of *U*(*r*) by the Coulomb potential (Eq. 3) may be valid in low ionic strength environments, it significantly overestimates the potential between the ions in solutions with a high ionic strength, *I* [83, 84]. In a solution with a large electrolyte concentration, such as in ionic liquids, inert counterions preferably surround the ions of the ion pair, thus screening the Coulomb potential acting between them. The top of Fig. 8 displays a simulation of $k_{{\text {sep}}}$ in ACN as a function of *I*. It is based on the Eigen equation with a potential *U*(*r*) calculated using Debye-Hückel theory [83–86] (see SI section S11 for details). The increase of $k_{{\text {sep}}}$ with ionic strength can be attributed to the reduced Coulombic attraction between the ions via a shielding effect.

The decrease of the intrapair electrostatic potential upon increasing *I*, rises the energy of the ion pair and, thus, the driving force for CR. As CR of the $\hbox {Pe}^{\cdot +}$/$\hbox {Pe}^{\cdot -}$ pair is highly exergonic, it occurs in the inverted regime. Consequently, increasing ionic strength and the CR driving force should result in a slowing down of charge recombination [69]. Figure 8 (top) shows a simulation^3^ of $k_{{\text {CR}}}$ as a function of *I* calculated using the semi-classical Marcus expression for the ET rate constant (see SI section S11 for details).

This figure illustrates the opposite effect of the ionic strength on $k_{{\text {sep}}}$ and $k_{{\text {CR}}}$. Taking both effects into account results in the ionic strength dependence of $\phi _{{\text {sep}}}$ shown in the bottom of Fig. 8. While the assumptions made in our calculations may be simplistic, this simulation nevertheless demonstrates the qualitative change in $\phi _{{\text {sep}}}$ with respect to *I*. It highlights that a higher ionic strength is generally in favor of a larger cage-escape yield for photoinduced ET reactions with Coulombic attraction in the ensuing ion pair. Indeed, such a salt effect has been known for a long time [69, 87–89]. Grosso et al. [89] observed for instance that the dissociation of the pyrene/*N,N*-dimethylaniline exciplex into free ions could be induced upon addition of inert salts in non-polar solvents. Similarly, Rosspeintner et al. [69] found an enhanced cage-escape yield in the photoinduced ET between Pe and *N,N*-dimethylaniline in RTILs compared to dipolar solvents of the same viscosity.

## Conclusion

We investigated the excited state dynamics of Pe at high concentration in six solvents of different polarity. We observed self-quenching and the formation of the excimer in all solvents including highly polar ones. However, in the latter media, free ions resulting from symmetry-breaking charge separation are also generated. The time evolution of the different intermediates is better reproduced assuming two competitive quenching pathways, SB-CS and excimer formation. By contrast to the exciplexes encountered in photoinduced ET between chemically distinct donors and acceptors, the dissociation of the excimer to free ions does not act a gateway towards free ions but should rather be seen as an unproductive quenching channel. Contrary to intramolecular SB-CS, excimer formation cannot be suppressed by preventing large coupling between the reactants with an adequate linker. Our results with RTILs suggest that the large effective polarity combined with the high viscosity favour SB-CS over excimer formation. This is due to the fact that SB-CS, if sufficiently exergonic, can occur at broader range of distances/orientation compared to excimer formation. RTILs offer the additional advantage over conventional dipolar solvents to facilitate the dissociation of the ion pairs into free ions by screening the intrapair electrostatic potential. The observed charge-shielding effect in RTILs opens up new possibilities for achieving efficient charge separation. However, further systematic investigations are required to fully understand and characterize this effect and its implications for bimolecular photoinduced electron transfer reactions.

### Supplementary Information

The Supporting Information associated with this manuscript contains the following files:

- Supporting_Information: influence of photoionisation, solvent properties, additional data, calculation of the free ion yields, triplet sensitisation experiments, simulation of the effect of ionic strength on $\phi _{\textrm{sep}}$),
- ns_TA [MP4]: movie illustrating the time dependence of the transient absorption spectra.

Below is the link to the electronic supplementary material.

Supplementary file 1 (pdf 2433 KB)

Supplementary file 2 (MP4 2847 KB)

## Data Availability

All data cabe downloaded from https://doi.org/10/gs79xd.

## Footnotes

1 Note that the time constants obtained from global analysis should be considered with caution. Although the experiment was carried out in front-face geometry the LE emission still shows the signs of the reabsorption effect which is known to influence the lifetimes in this region [43]. Furthermore, as excimer formation is a bimolecular process, an exponential model cannot accurately reproduce the data.

2 The transformation of the LE spectrum to the excimer and then to the ion bands in polar solvents, as well as the simple decay of the excimer in non-polar solvents, can be especially appreciated by watching a video of the time-dependent TA spectra provided in the supplementary Material. In the video, the y-scale of each spectrum is automatically adjusted to the absorption maximum, allowing for clear visualization of the spectral changes.

3 Note that we did not incorporate any ionic strength dependence on the reorganization energy as its change with ionic strength is expected to be small [69].
