# Supplementary material for: Bimolecular photoinduced symmetry-breaking charge separation of perylene in solution
Source: Photochem Photobiol Sci. 2023 Dec 22;23(1):93–105. doi: 10.1007/s43630-023-00504-3 (PMC10834668; doi:10.1007/s43630-023-00504-3)
Supplement: Supplementary file 1 — (pdf 2433 KB) [file 43630_2023_504_MOESM1_ESM.pdf]

**SUPPORTING INFORMATION FOR:**

# Bimolecular Symmetry-Breaking Charge Separation of Perylene in Solution

Johannes Wega, Eric Vauthey[a]*

[a] *Department of Physical Chemistry, University of Geneva, CH-1205 Geneva, Switzerland.*

**Email:* johannes.wega@unige.ch
eric.vauthey@unige.ch

## Contents

**Page**

1. Influence of Photoionization . . . . . S2
2. Solvent Properties . . . . . S2
3. Absorption Spectra . . . . . S2
4. Excimer Emission . . . . . S3
5. Non-normalized Global Analysis Data for the Dipolar Solvents . . . . . S4
6. Transient Absorption Spectrum in BMIM . . . . . S4
7. Normalized Free Ion Yield . . . . . S5
   7.1. Calculation by Comparative Actinometry . . . . . S5
   7.2. Estimation of the Error . . . . . S7
8. Contribution of Static Quenching . . . . . S8
9. Triplet Sensitization Experiments . . . . . S9
   9.1. Bubbling with Air . . . . . S9
   9.2. Population through Photoinduced Electron Transfer . . . . . S10
10. Global Target Analysis . . . . . S11
11. Simulation of the Ionic Strength Dependence of $\phi_{sep}$ . . . . . S12
12. References . . . . . S13

# 1. Influence of Photoionization

Konuk *et al.*[1] observed photoionization of Pe at 355 nm excitation in flash photolysis experiments (Pe $\xrightarrow{h\nu}$ Pe* $\xrightarrow{h\nu}$ Pe** $\longrightarrow$ $Pe^{\bullet+} + e^-$) at 120 mJ per excitation pulse. Assuming a typical beam diameter of 5 mm of an Nd:YAG laser used in flash photolysis experiments, results in a fluence of 1.2 $J\,cm^{-2}$. As we do not observe the formation of $Pe^{\bullet+}$ in the non-polar solvents taken together with the much lower fluence used in our experiments, we do not attribute the appearance of ions in polar solvents to photoionization.

# 2. Solvent Properties

Characteristic solvent properties of the solvents used here are summarized in Table **S1**. The dielectric constant $\varepsilon$, refractive index $n_D$ and viscosity $\eta$ of each solvent at 20 °C were taken from literature[2]

The diffusion rate constant was approximated by:[3]

$$k_{\mathrm{diff}} = \frac{8RT}{3\eta} \tag{s1}$$

with $R$ being the ideal gas constant and $T$ the temperature (taken here as 293.15 K).

The orientational polarizability or Onsager function[4], a direct measure of solvent polarity, was calculated using:

$$\Delta f = \frac{2(\varepsilon_r - 1)}{2\varepsilon_r + 1} - \frac{2\left(n_D^2 - 1\right)}{2n_D^2 + 1} \tag{s2}$$

The same set of properties for the used RTIL is presented in Tab. **S2**.

TABLE **S1**: Dielectric constant $\varepsilon$, refractive index $n_D$, viscosity $\eta$, orientational polarizability $\Delta f$ as well as diffusion rate constant $k_{\mathrm{diff}}$ at 20 °C for the different dielectric solvents used. a) Taken from Ref. 2.

| solvent | $\varepsilon_r$ $^{(a)}$ | $n_D$ $^{(a)}$ | $\eta$/cP $^{(a)}$ | $\Delta f$ | $k_{\mathrm{diff}}$ /$M^{-1}$ $s^{-1}$ |
|---|---|---|---|---|---|
| TOL | 2.41 | 1.497 | 0.57 | 0.032 | $1.14 \times 10^{10}$ |
| CHF | 4.98 | 1.445 | 0.55 | 0.306 | $1.18 \times 10^{10}$ |
| THF | 7.73 | 1.407 | 0.47 | 0.423 | $1.37 \times 10^{10}$ |
| ACO | 21.05 | 1.359 | 0.31 | 0.570 | $2.11 \times 10^{10}$ |
| ACN | 36.70 | 1.343 | 0.35 | 0.610 | $1.87 \times 10^{10}$ |
| DMSO | 46.45 | 1.479 | 2.20 | 0.526 | $2.96 \times 10^{9}$ |

TABLE **S2**: Dielectric constant $\varepsilon$, refractive index $n_D$, viscosity $\eta$, orientational polarizability $\Delta f$ as well as diffusion rate constant $k_{\mathrm{diff}}$ at 20 °C for the different RTIL used. a) Taken from Ref. 5. b) Taken from Ref. **?** . c) Taken from Ref. 6. d) Taken from Ref. 7. e) Taken from Ref. 8.

| RTIL | $\varepsilon_r$ | $n_D$ | $\eta$/cP | $\Delta f$ | $k_{\mathrm{diff}}$/$M^{-1}$ $s^{-1}$ |
|---|---|---|---|---|---|
| EMIDCA | 11.0[(a)] | 1.511[(b)] | 22.8[(b)] | 0.406 | $2.85 \times 10^{8}$ |
| BMIM | 12.2[(c)] | 1.426[(d)] | 135.6[(e)] | 0.474 | $4.79 \times 10^{7}$ |

# 3. Absorption Spectra

Figure **S1** illustrates the comparison between the normalized steady-state absorption spectra of the diluted solutions, measured in a 1 cm quartz cuvette with concentrations in the µM regime, and the absorption spectra of the saturated solutions with concentrations in the mM regime measured in the 10 µm cuvette for each solvent.

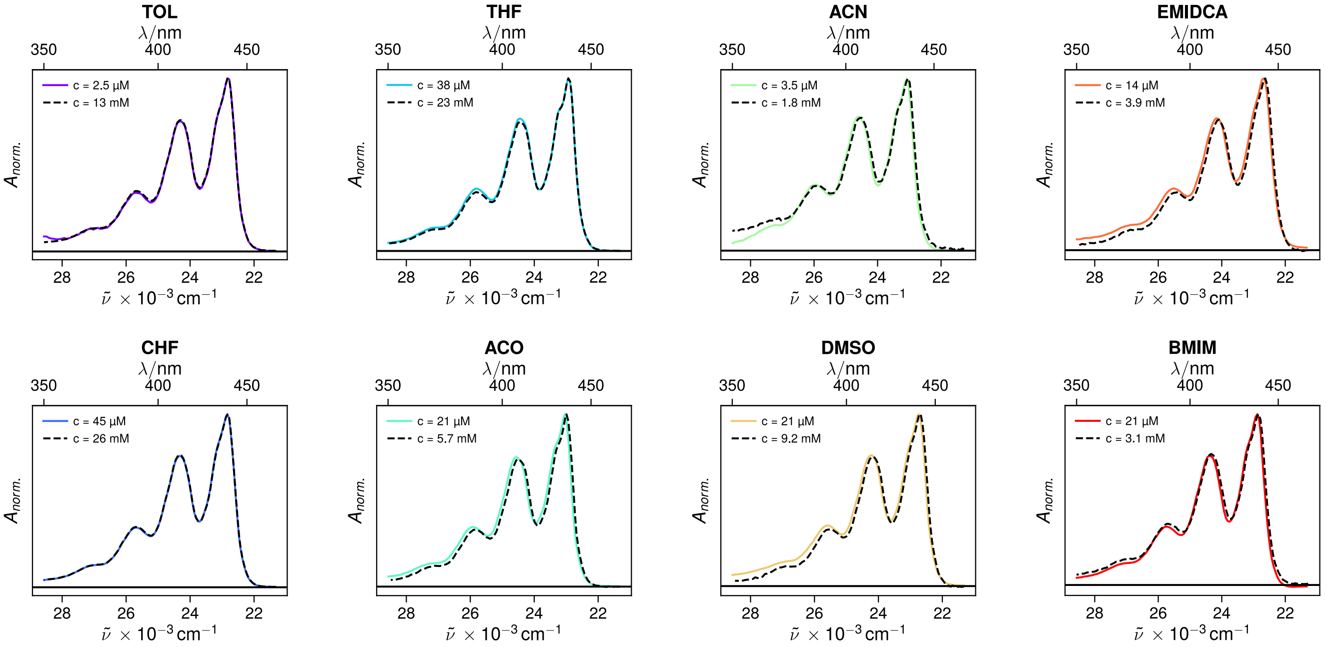


**Figure S1**: Steady-state of absorption spectra of dilute (solid line) and concentrated (dashed line) solutions of perylene in the different solvents used in this study.

# 4. Excimer Emission

Figure **S2** shows the emission spectra of dilute and concentrated Pe solutions in the weakly polar solvent TOL, the medium polar solvent THF and the polar solvent DMSO. From the concentrated and dilute spectrum in each solvent, the excimer emission spectrum (see Fig. **S2** bottom) could be extracted as explained in the main text.

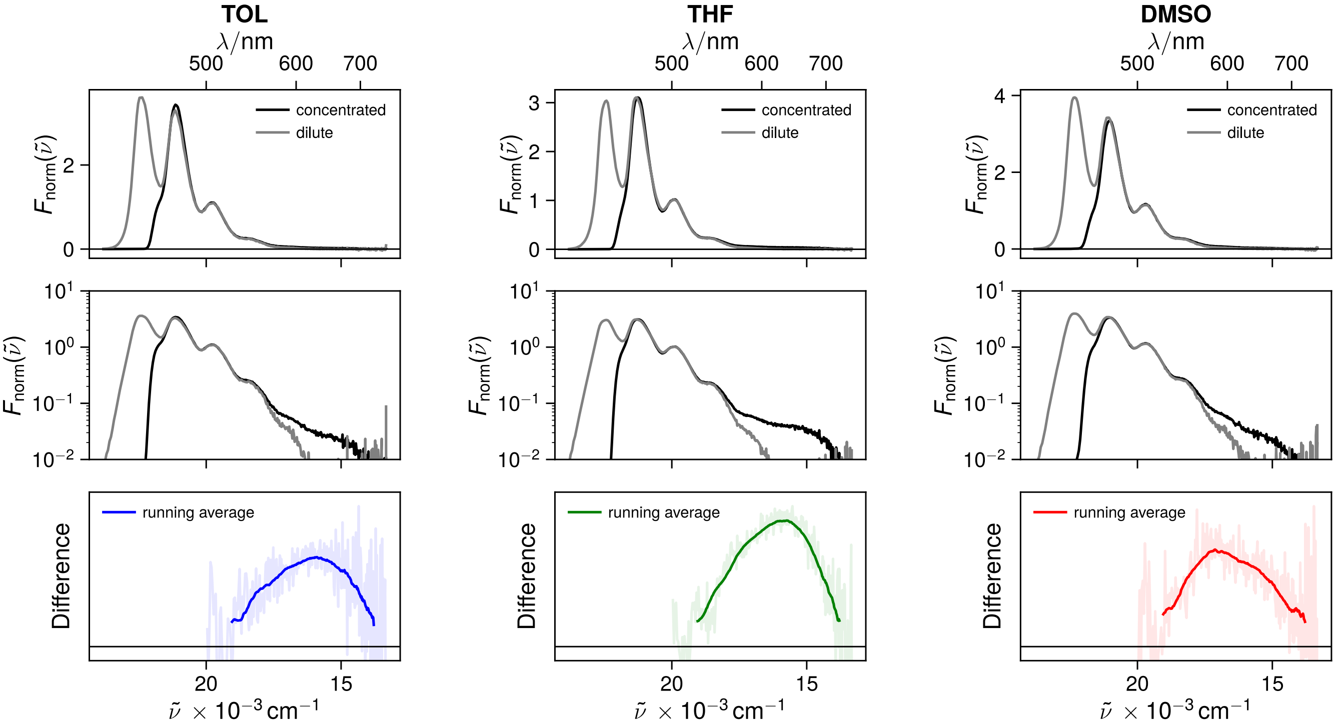


**Figure S2**: Steady-state emission spectra of dilute (gray line) and concentrated (black line) Pe solutions on a linear (top) and logarithmic (middle) scale in TOL (left), THF (middle) and DMSO (right) normalized at 500 nm. The bottom panels show the difference between the concentrated and dilute spectrum above 500 nm (transparent line). A 50-point moving average (solid line) highlights the excimer emission peak observed in the different solvents.

# 5. Non-normalized Global Analysis Data for the Dipolar Solvents

The results of the global analysis of the TA of the concentrated Pe solutions for all dipolar solvents is shown in Fig. **S3**. The data highlights the small signal amplitude of the ion and triplet peaks in comparison to the inital TA of the excited state. It also shows that the amount of triplet formed in each solvent is similar.

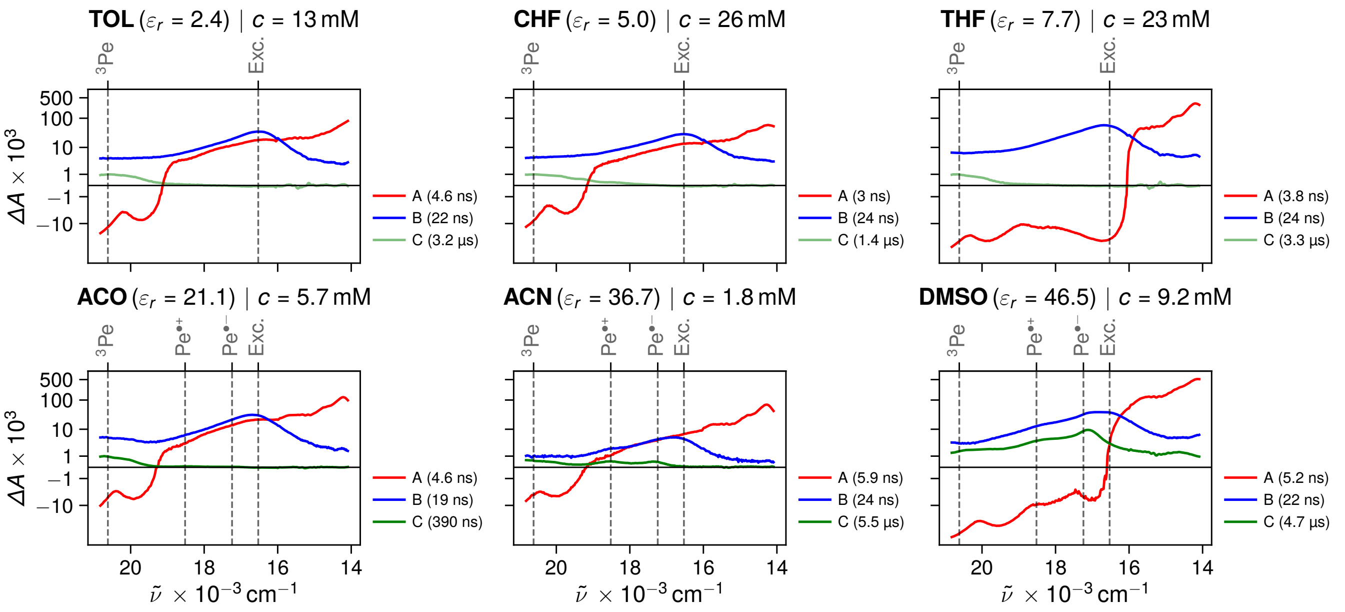


**Figure S3**: Non-normalized version of the global analysis data shown in Fig. 4 in the main text. An arcsinh scale is used for the y-axis in order to ease comparison between the large A and the small C spectrum.

# 6. Transient Absorption Spectrum in BMIM

The TA spectra measured with a concentrated Pe solution in the RTIL 1-butyl-3-methylimidazolium tetrafluoroborate (BMIM, Fig. **S4** A) are shown in Fig. **S4** B. Global analysis of the TA data clearly shows the presence of free ions (Fig. **S4** B) despite the high viscosity and subsequently slow diffusion rate in BMIM (see Table **S2**). The observation of ions can nonetheless be justified by considering that the diffusion rate in highly viscous RTIL has not reached its stationary limit during the lifetime of the Pe excited state and the actual quenching rate constant is much higher than the diffusion rate calculated by Eq. s1 (see section 5.1).

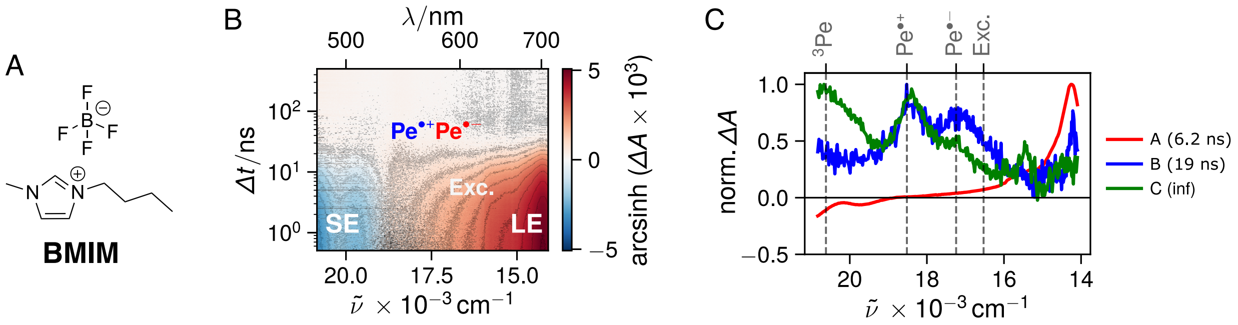


**Figure S4**: A) Chemical structure of the RTIL BMIM. B) Contour plot of the nanosecond TA-spectrum of Pe in BMIM. C) Normalized evolution associated difference spectra (EADS) obtained from global analysis of the transient absorption spectrum assuming a sequential model A → B → C.

# 7. Normalized Free Ion Yield

## 7.1 Calculation by Comparative Actinometry

The normalized free ion yield $\phi^n_{FI}$ is defined as the the quantum yield of free ions $\phi_{FI}$ normalized with respect to the quenching efficiency $\phi_q$ :[9–12]

$$\phi^n_{FI} = \frac{\phi_{FI}}{\phi_q} \tag{s3}$$

The free ion yield may thereby be obtained from the concentration of excited states [Pe*] generated by the laser pulse with respect to the concentration of free ions $[Pe^{\bullet+/-}]$:

$$\phi_{FI} = \frac{[Pe^{\bullet+}]}{[Pe^*]} = \frac{[Pe^{\bullet-}]}{[Pe^*]} \tag{s4}$$

In order to determine the initial concentration of Pe* generated by the excitation pulse we employ comparative actinometry using $Ru(bipy)_3^{2+}$ in DMSO. In brief, by performing a measurement using the exact same experimental configuration using $Ru(bipy)_3^{2+}$ in DMSO it is possible to determine the initial concentration of excited $Ru(bipy)_3^{2+}$:

$$[Ru^*] = \frac{\Delta A_{Ru}}{\Delta \varepsilon_{Ru} l} \tag{s5}$$

where $\Delta A_{Ru}$ is the transient absorption signal at 448 nm at time-zero, $l$ the pathlength of the cuvette (here 1 mm) and $\Delta\varepsilon_{Ru} = (-11\,300 \pm 1700)\,M^{-1}cm^{-1}$.[13] $Ru(bipy)_3^{2+}$ is chosen as an actinometer here as its differential extinction coefficients with respect to its $^3$MLCT excited state have been accurately determined for the use of actinometric experiments in transient absorption spectroscopy.[13] Moreover, the long microsecond lifetime of its $^3$MLCT which is populated quickly from the $^1$MLCT on a subpicosecond timescale ensures that the intial transient absorption at the beginning of the experiments (at around 0.3 ns) has not significantly decayed yet. Knowing the initial concentration of excited actinometer, the concentration of Pe* can be calculated using:

$$[Pe^*] = [Ru^*] \cdot \underbrace{\frac{1-10^{-A_{Pe}}}{1-10^{-A_{Ru}}}}_{1/\mathrm{corr}} \tag{s6}$$

whereby corr accounts for the difference in absorbance of the actinometer and sample solution with $A_{Ru}$ and $A_{Pe}$ being the steady-state absorbances at the excitation wavelength.

The concentration of free charge separated products may be obtained by using the transient absorption at 538 nm where $Pe^{\bullet+}$ has its absorption maximum.[14,15] It has been shown previously that the ion signals decay on the sub-50 ns timescale[16] attributed to the recombination of weakly coupled ion pairs. Furthermore, the broad absorption peak of the Pe excimer[17] which has a lifetime of approximately 20 ns (see global analysis results) leads to a transient absorption on top of the ion absorption. Keeping these two factors in mind together with the fact that free ion yields are generally determined using flash photolysis using the plateau value of the ion signal after around 100 ns, we used the transient absorbance of the ion signal at its initial plateau value at 100 ns, $\Delta A_{Pe^{\bullet+}}$, after the excimer has decayed and weakly coupled ion pairs have recombined:

$$[Pe^{\bullet+}] = \frac{\Delta A_{Pe^{\bullet+}}}{\Delta \varepsilon_{Pe^{\bullet+}} l} \tag{s7}$$

with $\Delta\varepsilon_{Pe^{\bullet+}} = (55\,600 \pm 5000)\,M^{-1}cm^{-1}$ being the extinction coefficient of $Pe^{\bullet+}$ at 538 nm.[14] The kinetic traces of the sample and actinometer solutions as well as the corresponding transient absorption values used for the analysis in the solvents where ion signals were observed are shown in Fig. **S5**.

Taking together Eq. s4, Eq. s5, Eq. s6 and Eq. s7, the yield of charge separation may be calculated by:[18–21]

$$\phi_{FI} = \frac{\Delta A_{Pe^{\bullet+}}}{\Delta \varepsilon_{Pe^{\bullet+}}} \cdot \frac{\Delta \varepsilon_{Ru}}{\Delta A_{Ru}} \cdot \mathrm{corr} \tag{s8}$$

Previous experiments by Katoh *et al.*[17] showed that excimer formation in concentrated Pe solution takes place with quenching rate close to the diffusion rate in dielectric solvents. Therefore, we estimate the fraction of excited state quenched using:

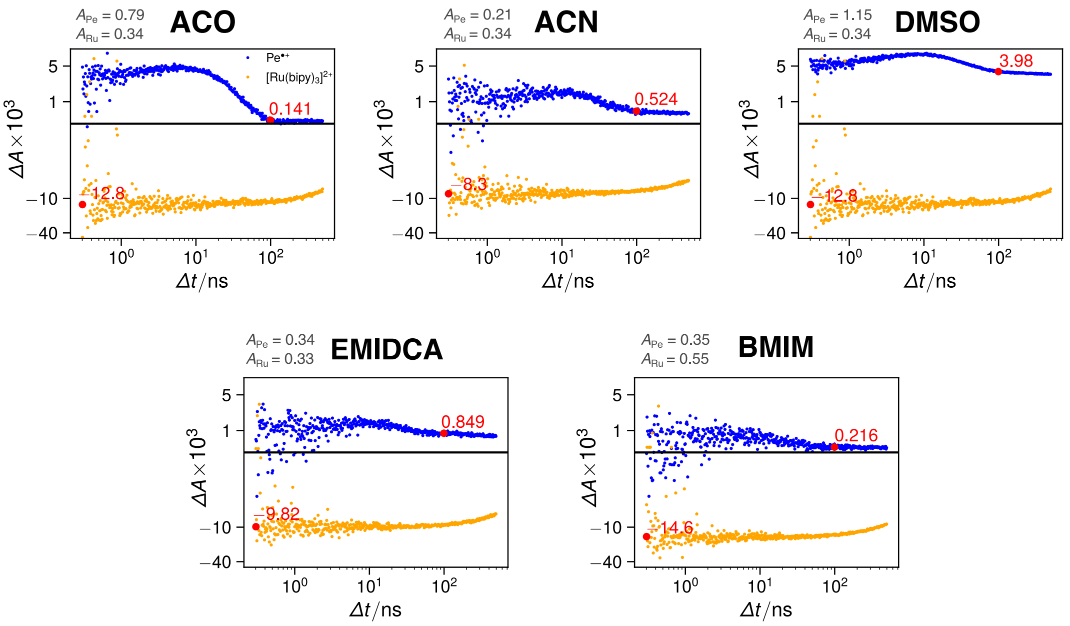


**Figure S5**: Kinetic traces of the TA signal at 538 nm (blue, absorption maximum[14,15] of $Pe^{\bullet+}$) in the different polar solvents / RTIL used together with the TA trace at 448 nm of a solution of $Ru(bipy)_3^{2+}$ measured under identical experimental configuration used for the actinometry. Numerical values used for the analysis are highlighted in each plot.

$$\phi_q = 1 - \left(1 + k_q \tau_0 c\right)^{-1} \tag{s9}$$

whereby we assume $k_q \approx k_{diff}$ with $c$ being the concentration of the solution and $\tau_0$ the excited state lifetime of Pe at low concentration which we take as $(5.0 \pm 0.5)$ ns. While the assumption $k_q \approx k_{diff}$ might be justified for the low-viscosity dielectric solvents, it would result in a significant overestimation of the free ion yield in the highly viscous ionic liquids. Rosspeintner *et al.*[22,23] could show that electron transfer in ionic liquids does not proceed faster than diffusion, as erroneously assumed before by a larger quenching rate constant $k_q$ commonly determined by Stern-Volmer plots than the diffusion rate constant calculated by Eq. s1. The discrepancy arises from fact that the diffusion rate never reaches its stationary regime in high viscosity liquids during the lifetime of the Pe excited state. The flux of the reactants towards each other strongly depends on the distance between them. In the beginning reactants that are already close to each other will diffuse together with a larger rate and only as time increases will the flux of the reactants towards each other approach a steady-state.[3] Consequently, as the viscosity is increased the time needed to reach this stationary regime will also increase. This can be illustrated by the simple Smoluchowski diffusion rate constant:[3]

$$k_{smolu}(t) = 8\pi\sigma D\left(1 + \frac{\sigma}{\sqrt{2\pi D t}}\right) \tag{s10}$$

for two spherical reactants with radius $r$ with the same diffusion coefficient:[3]

$$D = \frac{k_B T}{6\pi r \eta} \tag{s11}$$

In the simple Smoluchowski model, it is assumed that once the reactants are brought together by diffusion and once they have reached the contact distance $\sigma$ they react immediately. A simulation of the time-dependent diffusion rate constant for ACN, DMSO and the two RTIL used assuming $\sigma = 2r$ is shown in Fig. **S6**. The simulation highlights that during the lifetime of the Pe excited state the effective diffusion rate constant in the RTIL is much higher than its stationary value and only approaches $k_{diff}$ on the micro-millisecond timescale. Only as $t \to \infty$, Eq. s10 converges to Eq. s1. On the other hand, the assumption $k_q \approx k_{diff}$ is less problematic for the low-viscosity dielectric solvents as the stationary regime is reached much quicker within the excited state lifetime.

In order to account for this effect, we approximate the quenching rate as the Smoluchowski diffusion rate constant at the beginning of the experiment at 300 ps. The results obtained from the comparative actinometry are summarized in Table **S3**.

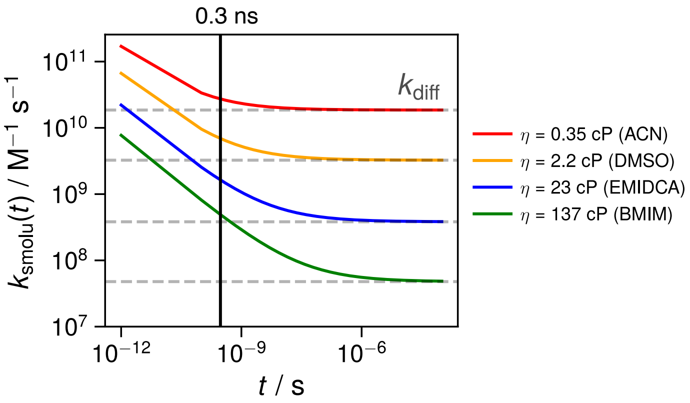


**Figure S6**: Simulation of the time dependence of the simple Smoluchowski diffusion rate constant assuming $r = 4\,\text{Å}, \sigma = 2r$ for ACN, DMSO and the two RTIL used. The simulation highlights that at the start of the experiment (at around 0.3 ns) the diffusion rate is much larger than the stationary limit $k_{\text{diff}}$ for the ionic liquids.

TABLE **S3**: Summary of results obtained for the different solvents / RTIL used.

| solvent | $c$/mM | $k_q/10^9\,\text{M}^{-1}\text{s}^{-1}$ | $\phi_q$/% | $\phi_{\text{FI}}$/% | $\phi^n_{\text{FI}}$/% |
|---|---|---|---|---|---|
| ACO | $5.7 \pm 0.6$ | $30 \pm 5$ | $46 \pm 6$ | $0.12 \pm 0.11$ | $0.3 \pm 0.2$ |
| ACN | $1.4 \pm 0.2$ | $27 \pm 5$ | $16 \pm 3$ | $1.7 \pm 0.6$ | $11 \pm 4$ |
| DMSO | $9.0 \pm 1.0$ | $6.9 \pm 0.5$ | $24 \pm 3$ | $3.8 \pm 0.8$ | $16 \pm 4$ |
| EMIDCA | $3.9 \pm 1.0$ | $1.6 \pm 0.5$ | $3 \pm 1$ | $1.7 \pm 0.4$ | $55 \pm 23$ |
| BMIM | $3.1 \pm 0.3$ | $0.49 \pm 0.05$ | $0.8 \pm 0.1$ | $0.4 \pm 0.2$ | $46 \pm 26$ |

## 7.2 Estimation of the Error

Taken the many assumptions used to assess the free ion yield together with the problems of a formal kinetic approach for bimolecular photoinduced electron transfer reactions[24,25] the numerical values of the free ion yields reported here should be taken with caution. The calculations, however, highlight nonetheless an increase of the free ion yield as the polarity of the solvent is increased as well as an increased escape of ions in the RTIL compared to the dielectric solvents. In order to assess whether these differences are significant we performed a simple error calculation.

The error $\text{err}_Y$ of a quantity $Y$, dependent on $i,\ldots,M$ independent variables $x_i,\ldots,x_M$ may be calculated by error propagation:

$$\text{err}_Y = \sqrt{\sum_{i=1}^{M}\left(\frac{\partial Y}{\partial x_i}\right)^2 \text{err}^2_{x_i}} \tag{s12}$$

whereby $\text{err}_{x_i}$ accounts for the errors of the individual variables.

The error of the normalized free ion yield therefore depends on the individual errors of the charge separation yield and the fraction of excited states quenched:

$$\text{err}_{\phi^n_{\text{FI}}} = \sqrt{\left(\frac{\text{err}_{\phi_{\text{FI}}}}{\phi_q}\right)^2 + \left(\frac{\phi_{\text{FI}} \cdot \text{err}_{\phi_q}}{\phi_q^2}\right)^2} \tag{s13}$$

Let us first determine the uncertainty in the fraction of excited states quenched. Examining Eq. s9 shows that $\phi_q$ is subject to three variables, namely the concentration $c$, the quenching rate $k_q$ as well as the excited state lifetime at infinite dilution. Hence its error is given by:

$$\text{err}_{\phi_q} = \sqrt{\frac{(\text{err}^2_c c^2 k_q^2 + (c^2 \text{err}^2_{k_q} + \text{err}^2_{\tau_0} k_q^2)\tau_0^2)}{(1 + c k_q \tau_0)^4}} \tag{s14}$$

We determined the concentration used in the experiments photometrically assuming an extinction coefficient of $\varepsilon_{\text{Pe}} \approx (38\,500 \pm 4000)\,\text{M}^{-1}\text{cm}^{-1}$ at the absorption maximum in all solvents used. Taking Lambert-Beer's Law, assuming that the

extinction coefficient to be the largest contributor of the overall error in the concentration shows that the uncertainty in concentration is given by:

$$\mathrm{err}_c = c\,\frac{\mathrm{err}_{\varepsilon_{\mathrm{Pe}}}}{\varepsilon_{\mathrm{Pe}}} \tag{s15}$$

For the lifetime in absence of quencher we assume a value of $\tau_0 = (5.0 \pm 0.5)$ ns consistent with the lifetime of the Pe excited state in polar solvents. The quenching rate constant most likely accounts for the largest error in $\phi_\mathrm{q}$ due to the assumptions in its estimation. We therefore take an error of $0.5^n$ M$^{-1}$ s$^{-1}$ where $n$ is the exponent of the rate constant calculated in the specific solvent.

As indicated by Eq. s8 the charge separation yield is also subject to many individual errors. Firstly, in order to account for any sample evaporation during the actinometry we calculate error of the correction factor using:

$$\mathrm{err}_{\mathrm{corr}} = \sqrt{\frac{10^{-2A_{\mathrm{Pe}}}(1-10^{-A_{\mathrm{Ru}}})^2\mathrm{err}_A^2 \ln 10^2}{(1-10^{-A_{\mathrm{Pe}}})^4} + \frac{10^{-2A_{\mathrm{Ru}}}\mathrm{err}_A^2 \ln 10^2}{(1-10^{-A_{\mathrm{Pe}}})^2}} \tag{s16}$$

whereby we take $\mathrm{err}_A = 0.05$ as estimated by the difference in the absorbance prior and after the TA experiment typically observed in the measurements. Taking this error into account the uncertainty in the charge separation yield becomes:

$$\mathrm{err}_{\phi_{\mathrm{FI}}} = \phi_{\mathrm{FI}}\sqrt{\left(\frac{\mathrm{err}_{\Delta A}}{\Delta A_{\mathrm{Pe}^{\bullet+}}}\right)^2 + \left(\frac{\mathrm{err}_{\Delta\varepsilon_{\mathrm{Pe}^{\bullet+}}}}{\Delta\varepsilon_{\mathrm{Pe}^{\bullet+}}}\right)^2 + \left(\frac{\mathrm{err}_{\Delta\varepsilon_{\mathrm{Ru}}}}{\Delta\varepsilon_{\mathrm{Ru}}}\right)^2 + \left(\frac{\mathrm{err}_{\Delta A}}{\Delta A_{\mathrm{Ru}}}\right)^2 + \left(\frac{\mathrm{err}_{\mathrm{corr}}}{\mathrm{corr}}\right)^2} \tag{s17}$$

where we assume the following errors for the extinction coefficients: $\mathrm{err}_{\Delta\varepsilon_{\mathrm{Pe}^{\bullet+}}} = 5000\,\mathrm{M^{-1}cm^{-1}}$, $\mathrm{err}_{\Delta\varepsilon_{\mathrm{Ru}}} = 1700\,\mathrm{M^{-1}cm^{-1}}$[13] as well as uncertainty of the TA signals of $\mathrm{err}_{\Delta A} = 0.1$ mOD. The obtained errors on all the relevant parameters are summarized together with the corresponding values in Table **S3**. Although the uncertainties are rather large, the calculation allows nonetheless to qualitatively address the increase in the free-ion yield as the solvent polarity is increased as well as the larger yield in the RTIL.

# 8. Contribution of Static Quenching

Next to the diffusional encounter of Pe* with a ground state Pe molecule to form the excimer and subsequently the ions in polar solvents, some of the initially excited molecules may already have another Pe molecule next to them such that excimer formation would happen directly without the need of diffusion. Here we demonstrate that this static contribution to the quenching is of minor importance for the concentrations employed in the experiments and that the assumption of a diffusion assisted excimer formation is reasonable.

Assuming molecules to be randomly distributed in the solution, the probability of $n$ Pe molecules to be found within a sphere of volume

$$V_\mathrm{S} = \frac{4}{3}\pi\sigma^3 \tag{s18}$$

with $\sigma$ being the contact distance needed for excimer formation may be estimated by Poisson statistics:[4,26]

$$P(n) = \frac{\lambda^n}{n!}e^{-\lambda} \tag{s19}$$

where

$$\lambda = V_\mathrm{S}[\mathrm{Pe}]N_\mathrm{A} \tag{s20}$$

with [Pe] being the concentration of the solution and $N_\mathrm{A}$ being Avogardro's constant. Taking, $\sigma = 8$ Å and a concentration of [Pe] = 10 mM shows that the probability to find one or more Pe next to Pe* at the moment of excitation in the quenching sphere is only about $P(n \geq 1) = 1 - P(0) = 1 - e^{-\lambda} \approx 1\,\%$. This static contribution may, however, play a role in the viscous RTIL.

# 9. Triplet Sensitization Experiments

## 9.1 Bubbling with Air

The triplet $^3(\pi,\pi^*)$-state of aromatic molecules exhibiting singlet $^1(\pi,\pi^*)$ excited states can be sensitized by molecular oxygen through energy transfer:

$$^{1*}\mathrm{Pe} + {}^3\mathrm{O}_2 \rightarrow {}^3\mathrm{Pe} + {}^1\mathrm{O}_2 \tag{s21}$$

resulting in the generation of singlet oxygen $^1O_2$.[9] This spin-allowed reaction which in principle can happen at every encounter is energetically feasible if the singlet-triplet energy gap of the molecule is larger than the singlet-triplet gap of molecular oxygen (0.86 eV).[9] Aromatic molecules exhibiting $(\pi,\pi^*)$ excited states like Pe often show larger singlet-triplet gaps in contrast to molecules with $(n,\pi^*)$-states.[9,27] Perylene possesses a singlet-triplet gap of approximately 1.3 eV[27], satisfying the energy criteria for triplet sensitization by molecular oxygen. On the other hand, $^3$Pe may also be populated through paramagnetic sensitization by triplet oxygen. The magnetic field induced by $^3O_2$ perturbs the Larmor frequencies of the two unpaired electrons in $^{1*}$Pe upon encounter resulting in dynamic interchange between singlet and triplet:[28]

$$^{1*}\mathrm{Pe} + {}^3\mathrm{O}_2 \rightleftharpoons {}^3\{{}^{1*}\mathrm{Pe}\cdots{}^3\mathrm{O}_2\} \xrightleftharpoons{\text{spin conversion}} {}^5\{{}^3\mathrm{Pe}\cdots{}^3\mathrm{O}_2\} \rightleftharpoons {}^3\mathrm{Pe} + {}^3\mathrm{O}_2 \tag{s22}$$

In an attempt to resolve the spectral features associated with the Pe-triplet state we performed TA measurements in solutions purged with air. Figure **S7** compares the TA spectra of concentrated Pe solution while bubbling the solutions with nitrogen and compressed air in ACN and DMSO. Indeed, triplet-sensitization is evident in both solvents by the appearance of a new positive TA at around 470-500 nm in good agreement with the triplet absorption spectrum of Pe reported in the literature.[25,27,29]

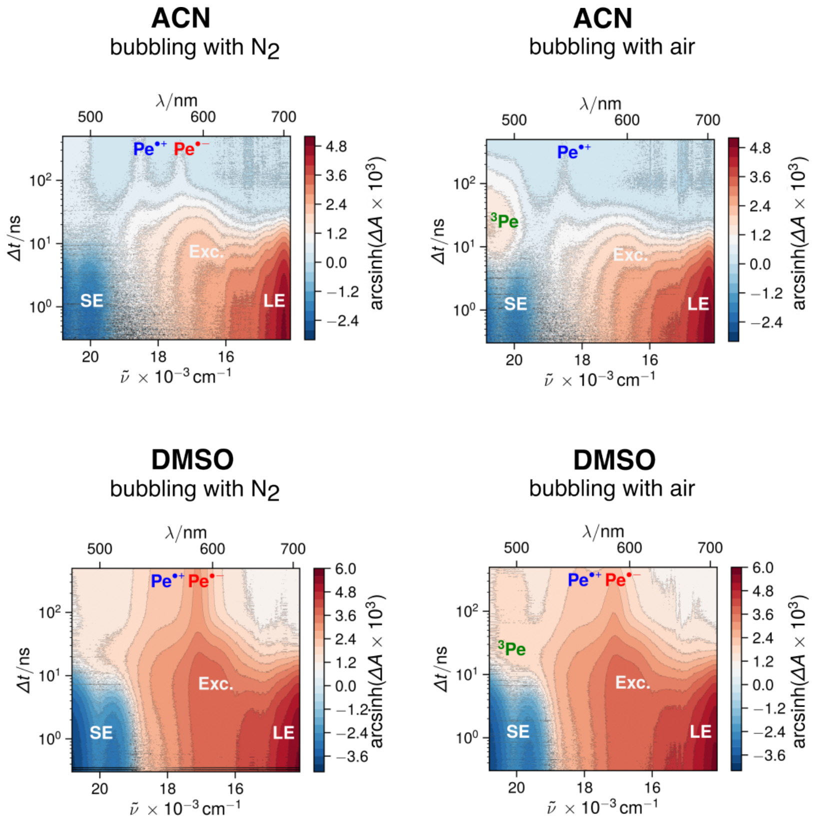


**Figure S7**: Comparison of transient absorption spectra of concentrated Pe solutions in ACN (top) and DMSO (bottom) while purging the solution with nitrogen (left) and compressed air (right).

Furthermore, it can be observed that the lifetime of the Pe-anion is significantly reduced in the aerated solutions. This may be due to oxidation by singlet/triplet oxygen to generate superoxide:

$$\begin{aligned} Pe^{\bullet-} + {}^3O_2 &\rightarrow Pe + {}^3O_2^{\bullet-} \\ \Delta G^0 &= -1.60\,eV \end{aligned} \tag{s23}$$

$$\begin{aligned} Pe^{\bullet-} + {}^1O_2 &\rightarrow Pe + {}^1O_2^{\bullet-} \\ \Delta G^0 &= -2.46\,eV \end{aligned} \tag{s24}$$

which seems energetically feasible taking into account the relevant reduction potentials: $E^0\left(Pe/Pe^{\bullet-}\right) = -1.66$ V vs. SCE[30], $E^0\left({}^1O_2/O_2^{\bullet-}\right) = +0.79$ V vs. SCE[9], $E^0\left({}^3O_2/O_2^{\bullet-}\right) = -0.60$ V vs. SCE[9]. This might also explain the different lifetimes of the Pe-anion and cation as well as their relative peak intensity ratios in the different solvents used. Although the solutions were purged with nitrogen during the measurements, we cannot completely exclude the presence of oxygen. As a result, some of the anion may undergo quenching, as evident by the shorter lifetime of $Pe^{\bullet-}$ compared to $Pe^{\bullet+}$ even in the de-aerated solution in for instance ACN.

The intensity of the $Pe^{\bullet+}$ band in DMSO is noticeably lower compared to that of $Pe^{\bullet-}$ band, despite their comparable extinction coefficients[25]. This difference could be attributed to the formation of Pe-dimer cation:

$$Pe^{\bullet+} + Pe \rightarrow Pe_2^{\bullet+} \tag{s25}$$

due to the higher CS-yield in DMSO. The absorption of the dimer cation is located around 500 nm[14] which may overlap with the triplet absorption band.

## 9.2 Population through Photoinduced Electron Transfer

Due to possible overlapping features of the excimer and ion signals (and any possible secondary products thereof) with the triplet absorption in TA spectrum of a Pe solution at high concentration, we implemented an alternative approach to extract the Pe triplet state absorption features. The main idea is illustrated in Fig. **S8**. Upon photoinduced CS between a donor (D) and acceptor (A), exemplary shown here for reductive quenching, the generated geminate ion pair initially produced as a singlet state ${}^1[{}^2A^- \cdots {}^2D^+]$ is known to be able to interconvert to a quasi-degenerate triplet ion state ${}^3[{}^2A^- \cdots {}^2D^+]$ by means of intersystem crossing (ISC) or hyperfine interactions.[16,31] This triplet ion pair then may recombine yielding the triplet state of the initially photoexcited species ${}^3[{}^3A \cdots {}^1D]$.

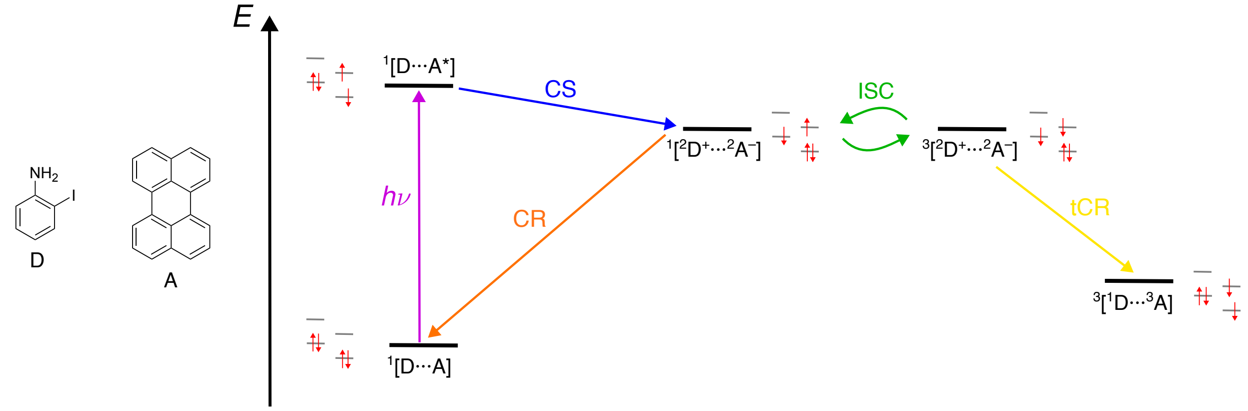


**Figure S8**: Energy-level scheme of a photoinduced electron transfer reaction resulting in a geminate ion pair after CS which can either recombine (CR) to the singlet ground state or can be converted to a triplet ion pair by intersystem crossing (ISC) which can recombine to the triplet state (tCR).

We tried to exploit this strategy to populate the triplet state of Pe through quenching with $m$-iodoaniline (see Fig. **S8**) with the idea of the heavy iodine atom in the quencher to accelerate ISC in the ion pair state.[32] We employed a dilute solution of Pe with high quencher concentration (90 mM) in the experiment. Using a dilute solution it was further possible to extend the spectral window to the ground state bleach (GSB) region. The resulting TA-spectrum is shown in Fig. **S9** A. Quenching is evident by a decreased excited state lifetime to roughly 3.0 ns. Next to the spectral features attributed to the GSB, SE and ESA new positive bands emerge at 450 - 480 nm and 400 - 420 nm after decay of the Pe excited singlet state. We do not observe the absorption features of ions generated upon photoinduced CS. This is likely due to the high quencher concentration which results primarily in the formation of strongly coupled ion pairs which recombine too quickly such that the free ion yield is too low in order to detect any transient absorption.

Analyzing the TA data globally using a sequential exponential decay model with two steps results in the evolution associated difference spectra (EADS) shown in Fig. **S9** B. EADS A shows the characteristic features of the Pe locally excited state (LE) absorption, SE and GSB. EADS B on the other hand, shows a combination of the GSB and new positive absorption bands which decay within around 1.7 µs and which we attribute to the triplet state of Pe. Comparison of the B spectrum with previously reported[25,29] triplet absorption spectra of Pe validates this assignment. Filling up EADS B with the GSB of Pe results in an approximate of the Pe-triplet absorption spectrum (red line in Fig. **S9** C). Next to the commonly cited[33–35] absorption peak at 480-485 nm three more absorption peaks at 455 nm, 420 nm and 400 nm become visible.

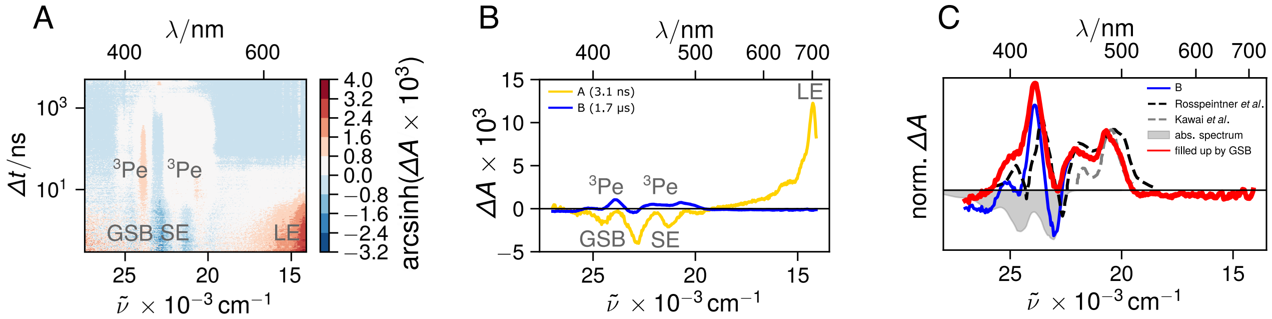


**Figure S9**: A) Nanosecond transient absorption spectrum of a dilute Pe solution containing 90 mM *m*-iodoaniline. B) Evolution associated difference spectra (EADS) obtained from global analysis of the transient absorption spectrum assuming a sequential model A → B → 0. C) Comparison of the B EADS with literature data[25,29] of the triplet absorption spectrum of Pe. The B-spectrum was filled up by the GSB in order to extract an approximate of the triplet absorption spectrum. Note that the data by Rosspeintner *et al.* represents the triplet-absorption spectra extracted from TA data prior to correcting the GSB in order to facilitate comparison with the B-spectrum.

## 10. Global Target Analysis

Fitting the transient absorption data in ACN, DMSO and the two ionic liquids assuming a branching type model from the LE to either the excimer or the ions (black box in Fig. **S10**) results in the species associated difference spectra (SADS) shown in Fig. **S10**.

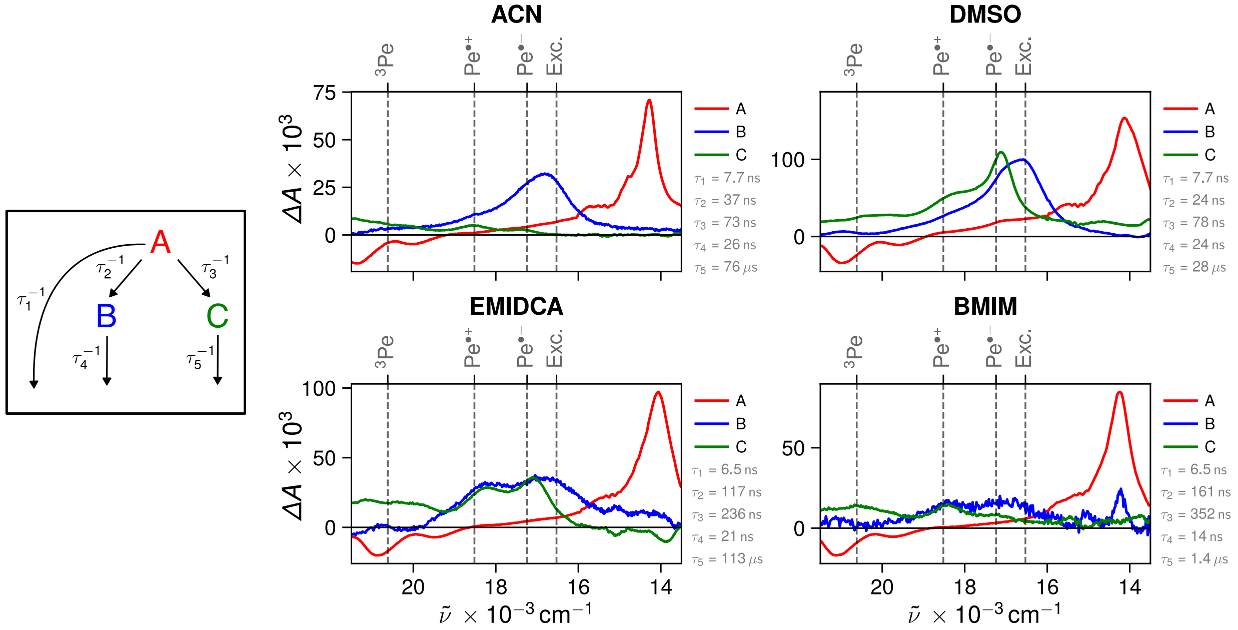


**Figure S10**: SADS obtained from global target analysis of the transient absorption data for the polar solvents ACN and DMSO as well as the two ionic liquids used using the kinetic model highlighted in the black box.

# 11. Simulation of the Ionic Strength Dependence of $\phi_{\text{sep}}$

As highlighted by Eq. 5, the ion separation yield $\phi_{\text{sep}}$ can be estimated from the rate constant of diffusional separation $k_{\text{sep}}$ and the rate constant for geminate charge recombination $k_{\text{CR}}$. The former, *i.e.* the steady-state diffusional escape rate contanst of an encounter pair, $\{\text{A}\cdots\text{B}\} \xrightarrow{k_{\text{sep}}} \text{A} + \text{B}$, of spherical species with radii $r_{\text{A}}$ and $r_{\text{B}}$, may be calculated using the Eigen-equation[21,36–40]:

$$k_{\text{sep}} = \frac{3(D_{\text{A}} + D_{\text{B}})}{\sigma^2} \exp\left(\frac{U(\sigma)}{k_{\text{B}} T}\right) \beta \tag{s26}$$

with

$$\beta^{-1} = \int_{\sigma}^{\infty} \frac{\exp\left(\frac{U(r)}{k_{\text{B}} T}\right)}{r^2} \, \mathrm{d}r \tag{s27}$$

where $D_{\text{A}}$ and $D_{\text{B}}$ are their diffusion coefficients which may be estimated using the Stokes-Einstein relation:[4]

$$D_{\text{i}} = \frac{k_{\text{B}} T}{6\pi r_{\text{i}} \eta} \tag{s28}$$

with $k_{\text{B}}$ being Boltzmann's constant, $\sigma$ the contact distance and $U(r)$ any existing potential between A and B. If A and B are ions and therefore charged this potential contribution may be approximated by the Coulomb potential (Eq. 2). While this approximation may be valid in low ionic strength solutions it significantly overestimates the potential between the ions in solutions with a high ionic strength.[21,40] In a solution with a large electrolyte concentration, such as in ionic liquids, inert counterions will preferably surround the ions of the ion pair thus screening the coulombic potential between them. The effective screened potential between A and B may be calculated using Debye-Hückel theory:[21,39–41]

$$U(r) = \frac{z_{\text{A}} z_{\text{B}} e^2}{4\pi \varepsilon_{\text{r}} \varepsilon_0 r} \left( \frac{1}{2} \left( \frac{\exp(\kappa r_{\text{A}})}{1 + \kappa r_{\text{A}}} + \frac{\exp(\kappa r_{\text{B}})}{1 + \kappa r_{\text{B}}} \right) \exp(-\kappa r) \right) \tag{s29}$$

with $z_{\text{A}}$ and $z_{\text{B}}$ being the charge numbers of A and B and

$$\kappa^{-1} = \sqrt{\frac{\varepsilon_{\text{r}} \varepsilon_0 k_{\text{B}} T}{2 e^2 N_{\text{A}} I}} \tag{s30}$$

the Debye screening length with $N_{\text{A}}$ being Avogadro's constant and $I$ the ionic strength of the solution. In the case of SB-CS, A = $\text{Pe}^{\bullet+}$, B = $\text{Pe}^{\bullet-}$ and $z_{\text{A}} = +1$, $z_{\text{B}} = -1$. For the computation of $k_{\text{sep}}$ we numerical integrate the Debye-Hückel potential, assuming radii of $r_{\text{Pe}^{\bullet+/-}} = 4\,\text{Å}$ (corresponding to the van der Waals volume of Pe) and a value of $\sigma = 8\,\text{Å}$. The viscosity of ACN is used in the simulation.

The rate of CR which also influences $\phi_{\text{sep}}$ was calculated using the Levich-Dogonadze semi-classical Marcus rate expression[42,43]

$$k_{\text{CR}} = \frac{2\pi}{\hbar} \frac{V^2}{\sqrt{4\pi \lambda_{\text{out}} k_{\text{B}} T}} \sum_{n=0}^{\infty} \frac{S^n}{n!} \exp(-S) \exp\left[ -\frac{\left(\Delta G^0_{\text{CR}} + n h \nu + \lambda_{\text{out}}\right)^2}{4 \lambda_{\text{out}} k_{\text{B}} T} \right] \tag{s31}$$

which specifically considers nuclear tunneling employing a single quantum mode with frequency $\nu$. This needs to be considered as CR is located in the Marcus-inverted region. In this equation, $V$ represents the electronic coupling and $S = \lambda_{\text{in}}/h\nu$ with $\lambda_{\text{in}}$ being the inner-sphere reorganization energy associated to the high-frequency mode. The outer-sphere reorganization energy $\lambda_{\text{out}}$ may be approximated by:[10,43]

$$\lambda_{\text{out}} = \frac{e^2}{4\pi \varepsilon_0} \cdot \left( \frac{1}{2 r_{\text{A}}} + \frac{1}{2 r_{\text{B}}} - \frac{1}{\sigma} \right) \cdot \left( \frac{1}{n_D^2} - \frac{1}{\varepsilon_{\text{r}}} \right) \tag{s32}$$

where $n_D$ is the refractive index of the solvent. The exergonicity of CR may be calculated using:[44]

$$\Delta G^0_{\text{CR}} = -e\left[E^0\left(\text{Pe}^{\bullet+}/\text{Pe}\right) - E^0\left(\text{Pe}/\text{Pe}^{\bullet-}\right)\right] - U(\sigma) \tag{s33}$$

For the computation of $k_{\text{CR}}$ we assumed typical values of, $V = 80\,\text{cm}^{-1}$, $\tilde{\nu} = 1500\,\text{cm}^{-1}$, $\lambda_{\text{in}} = 0.4\,\text{eV}$ and the solvent parameters of ACN.

# 12. References


(1) Konuk, R., Cornelisse, J. & McGlynn, S. Photoionization of perylene in polar solvents. *The Journal of chemical physics* **82**, 3929–3933 (1985).
(2) Marcus, Y. *The Properties of Solvents* No. v. 1 in The Properties of Solvents (Wiley, 1998).
(3) Henriksen, N. E. & Hansen, F. Y. *Theories of molecular reaction dynamics: the microscopic foundation of chemical kinetics* (Oxford University Press, 2018).
(4) Lakowicz, J. R. *Principles of fluorescence spectroscopy* (Springer, 2006).
(5) Hunger, J., Stoppa, A., Schrödle, S., Hefter, G. & Buchner, R. Temperature dependence of the dielectric properties and dynamics of ionic liquids. *ChemPhysChem* **10**, 723–733 (2009).
(6) Stoppa, A. *et al.* Interactions and dynamics in ionic liquids. *J. Phys. Chem. B* **112**, 4854–4858 (2008).
(7) Taib, M. M. & Murugesan, T. Density, refractive index, and excess properties of 1-butyl-3-methylimidazolium tetrafluoroborate with water and monoethanolamine. *J. Chem. Eng. Data* **57**, 120–126 (2012).
(8) Harris, K. R., Kanakubo, M. & Woolf, L. A. Temperature and pressure dependence of the viscosity of the ionic liquid 1-butyl-3-methylimidazolium tetrafluoroborate: viscosity and density relationships in ionic liquids. *J. Chem. Eng. Data* **52**, 2425–2430 (2007).
(9) Balzani, V., Ceroni, P. & Juris, A. *Photochemistry and photophysics: concepts, research, applications* (John Wiley & Sons, 2014).
(10) Bolton, J. R., Mataga, N. & McLendon, G. in *Introduction to electron transfer in inorganic, organic, and biological systems* (ACS Publications, 1991).
(11) Gilbert, A. & Baggott, J. E. *Essentials of molecular photochemistry* (1991).
(12) Gratzel, M. *Energy resources through photochemistry and catalysis* (Elsevier, 2012).
(13) Müller, P. & Brettel, K. [ru (bpy) 3] 2+ as a reference in transient absorption spectroscopy: differential absorption coefficients for formation of the long-lived 3 mlct excited state. *Photochem. Photobiol. Sci.* **11**, 632–636 (2012).
(14) Kimura, K., Yamazaki, T. & Katsumata, S. Dimerization of the perylene and tetracene radical cations and electronic absorption spectra of their dimers. *J. Phys. Chem.* **75**, 1768–1774 (1971).
(15) Shida, T. & Iwata, S. Electronic spectra of ion radicals and their molecular orbital interpretation. iii. aromatic hydrocarbons. *J. Am. Chem. Soc.* **95**, 3473–3483 (1973).
(16) Feskov, S. V. *et al.* Magnetic field effect on ion pair dynamics upon bimolecular photoinduced electron transfer in solution. *J. Chem. Phys.* **150**, 024501 (2019).
(17) Katoh, R., Sinha, S., Murata, S. & Tachiya, M. Origin of the stabilization energy of perylene excimer as studied by fluorescence and near-ir transient absorption spectroscopy. *J. Photochem. Photobiol., A* **145**, 23–34 (2001).
(18) Harriman, A., Porter, G. & Wilowska, A. Photoreduction of benzo-1, 4-quinone sensitised by metalloporphyrins. *J. Chem. Soc., Faraday Trans. 2* **79**, 807–816 (1983).
(19) Hoffman, M. Z. Cage escape yields from the quenching of excited tris (bipyridyl) ruthenium (2+) by methylviologen in aqueous solution. *J. Phys. Chem.* **92**, 3458–3464 (1988).
(20) Gardner, J. M., Abrahamsson, M., Farnum, B. H. & Meyer, G. J. Visible light generation of iodine atoms and i- i bonds: sensitized i-oxidation and i3- photodissociation. *J. Am. Chem. Soc.* **131**, 16206–16214 (2009).
(21) Aydogan, A. *et al.* Accessing photoredox transformations with an iron (iii) photosensitizer and green light. *J. Am. Chem. Soc.* **143**, 15661–15673 (2021).
(22) Rosspeintner, A., Koch, M., Angulo, G. & Vauthey, E. Salt effect in ion-pair dynamics after bimolecular photoinduced electron transfer in a room-temperature ionic liquid. *J. Phys. Chem. Lett.* **9**, 7015–7020 (2018).
(23) Koch, M., Rosspeintner, A., Angulo, G. & Vauthey, E. Bimolecular photoinduced electron transfer in imidazolium-based room-temperature ionic liquids is not faster than in conventional solvents. *J. Am. Chem. Soc.* **134**, 3729–3736 (2012).
(24) Rosspeintner, A. & Vauthey, E. Bimolecular photoinduced electron transfer reactions in liquids under the gaze of ultrafast spectroscopy. *Phys. Chem. Chem. Phys.* **16**, 25741–25754 (2014).
(25) Angulo, G., Rosspeintner, A., Lang, B. & Vauthey, E. Optical transient absorption experiments reveal the failure of formal kinetics in diffusion assisted electron transfer reactions. *Phys. Chem. Chem. Phys.* **20**, 25531–25546 (2018).
(26) Kaul, N. & Lomoth, R. The carbene cannibal: Photoinduced symmetry-breaking charge separation in an fe (iii) n-heterocyclic carbene. *J. Am. Chem. Soc.* **143**, 10816–10821 (2021).
(27) Birks, J. B. *Photophysics of Aromatic Molecules* Wiley monographs in chemical physics (Wiley, 1970).
(28) Turro, N. J., Ramamurthy, V., Scaiano, J. C. *et al. Modern molecular photochemistry of organic molecules* Vol. 188 (University Science Books Sausalito, CA, 2010).
(29) Kawai, K., Yamamoto, N. & Tsubomura, H. Simultaneous formation of perylene cation and anion by flash excitation of perylene in solutions. *Bull. Chem. Soc. Jpn.* **43**, 2266–2268 (1970).
(30) Parker, V. D. Energetics of electrode reactions. ii. the relationship between redox potentials, ionization potentials, electron affinities, and solvation energies of aromatic hydrocarbons. *J. Am. Chem. Soc.* **98**, 98–103 (1976).
(31) Schulten, K., Staerk, H., Weller, A., Werner, H.-J. & Nickel, B. Magnetic field dependence of the geminate recombination of radical ion pairs in polar solvents. *Z. Phys. Chem.* **101**, 371–390 (1976).
(32) Nicolet, O. & Vauthey, E. Heavy atom effect on the charge recombination dynamics of photogenerated geminate ion pairs. *J. Phys. Chem. A* **107**, 5894–5902 (2003).
(33) Zhang, Z., Ni, W., Ma, L., Sun, L. & Gurzadyan, G. G. Enhancement of singlet fission yield by hindering excimer formation in perylene film. *J. Phys. Chem. C* **126**, 396–403 (2021).
(34) Ni, W. *et al.* Singlet fission from upper excited electronic states of cofacial perylene dimer. *J. Phys. Chem. Lett.* **10**, 2428–2433 (2019).
(35) Ni, W., Sun, L. & Gurzadyan, G. G. Ultrafast spectroscopy reveals singlet fission, ionization and excimer formation in perylene film. *Sci. Rep.* **11**, 5220 (2021).
(36) Eigen, M. Über die kinetik sehr schnell verlaufender ionenreaktionen in wässeriger lösung. *Z. Phys. Chem.* **1**, 176–200 (1954).
(37) Steinfeld, J., Francisco, J. & Hase, W. Chemical kinetics and dynamics, upper saddle river, nj 1999.
(38) Hong, K. & Noolandi, J. Solution of the smoluchowski equation with a coulomb potential. i. general results. *J. Chem. Phys.* **68**,

5163–5171 (1978).
(39) Chiorboli, C., Scandola, F. & Kisch, H. Quenching of excited tris (2, 2'-bipyridine) ruthenium (ii) by metal 1, 2-dithiolene complexes. *J. Phys. Chem.* **90**, 2211–2215 (1986).
(40) Deetz, A. M., Troian-Gautier, L., Wehlin, S. A., Piechota, E. J. & Meyer, G. J. On the determination of halogen atom reduction potentials with photoredox catalysts. *J. Phys. Chem. A* **125**, 9355–9367 (2021).
(41) Huckel, E. & Debye, P. Zur theorie der elektrolyte. i. gefrierpunktserniedrigung und verwandte erscheinungen. *Phys. Z* **24**, 185–206 (1923).
(42) Levich, V. & Dogonadze, R. Adiabalic theory of electron transfer reactions, coll. ciech. *Chem. Commun., ïfi* 193–214 (1961).
(43) Marcus, R. Faraday symp. chem. soc., 10, 60, 1975;(h) marcus, ra. *Faraday Discuss. Chem. Soc* **74**, 7 (1982).
(44) Weller, A. Photoinduced electron transfer in solution: exciplex and radical ion pair formation free enthalpies and their solvent dependence. *Z. Phys. Chem.* **133**, 93–98 (1982).
